# First Case of a COVID-19 Patient Infected by Delta AY.4 with a Rare Deletion Leading to a N Gene Target Failure by a Specific Real Time PCR Assay: Novel Omicron VOC Might Be Doing Similar Scenario?

**DOI:** 10.3390/microorganisms10020268

**Published:** 2022-01-25

**Authors:** Mohammad Alkhatib, Maria Concetta Bellocchi, Greta Marchegiani, Sandro Grelli, Valeria Micheli, Daniele Stella, Bartolomeo Zerillo, Luca Carioti, Valentina Svicher, Paola Rogliani, Francesca Ceccherini-Silberstein

**Affiliations:** 1 Department of Experimental Medicine, University of Rome Tor Vergata, 00133 Rome, Italy; mohammad--alkhatib@hotmail.com (M.A.); mariac.bellocchi@gmail.com (M.C.B.); Gretamarchegiani@gmail.com (G.M.); grelli@med.uniroma2.it (S.G.); stelladaniele98@gmail.com (D.S.); luca.carioti@yahoo.com (L.C.); paola.rogliani@uniroma2.it (P.R.); 2 Virology Unit, Policlinico Tor Vergata, 00133 Rome, Italy; 3 Laboratory of Clinical Microbiology, Virology and Bioemergencies, ASST Fatebenefratelli Sacco L. Sacco Hospital, 20157 Milan, Italy; valeria.micheli@asst-fbf-sacco.it; 4 Respiratory Medicine Unit, Policlinico Tor Vergata, 00133 Rome, Italy; Bartolomeo.Zerillo@ptvonline.it; 5 Department of Biology, University of Rome Tor Vergata, 00133 Rome, Italy; valentina.svicher@uniroma2.it

**Keywords:** SARS-CoV-2, delta variant, omicron variant, N gene target failure, diagnostic assay, sequence, NGS, nucleocapsid deletion

## Abstract

Herein, we report a case of an Italian male infected by Delta sublineage AY.4 harboring an atypical deletion, leading to a N gene target failure (NGTF) by a commercial molecular assay for SARS-CoV-2 diagnosis (Allplex^TM^ SARS-CoV-2 Assay, Seegene). A 59-year-old unvaccinated patient was hospitalized for pulmonary embolism, with first negative results obtained by both molecular and antigen tests. After several days of viral negativity, he presented positive results for E and RdRP/S genes, but negative in N gene. Negativity in N gene was repeatedly confirmed in the following days. Suspecting an infection by the Omicron variant, SARS-CoV-2 genome sequencing was rapidly performed from nasopharyngeal swab by MiSeq and revealed the presence of the Delta sublineage AY.4 variant with an atypical deletion of six nucleotides, leading to G214-G215 deletion in the Nucleocapsid, thus responsible for NGTF. The analysis of GISAID sequences (N = 2,618,373 12 January 2022) showed that G214-G215 deletion is rarely occurring in most circulating Delta lineages and sublineages in the globe and Europe, with an overall prevalence never exceeding 0.2%. Hence, this study highlights the importance to perform SARS-CoV-2 sequencing and to characterize novel mutations/deletions that could jeopardize the proper interpretation of molecular diagnostic tests. Based on these assumptions, the role of deletions in the recently identified Omicron variant deserves further investigation.

## 1. Introduction

Since the beginning of the severe acute respiratory syndrome coronavirus 2 (SARS-CoV-2) pandemic, >22,000 viral amino acid mutations and >13,000 insertions/deletions have been spotted across the viral genome, with the potential to increase viral transmission, more severe disease, reduced effectiveness of treatments or vaccines, and/or diagnostic detection failures. The vast majority of mutations (73%) were in the ORF1ab, followed by 13% and 4% in the Spike and Nucleocapsid, respectively [1].

Starting from May 2021, a new variant, originally described in India, has become the predominant circulating variant of the COVID-19 pandemic. This variant of concern (VOC) was assigned as Delta by the World Health Organization and consists to date of 209 distinct sub-lineages (B.1.617.2 and AY.X, according to Pango phylogeny as 13 January 2022 [2]), that share L452R and T478K within the Spike and D63G, R203M, and D377Y in the Nucleocapsid [3]. The Delta variant has raised concerns due to its association with its high transmissibility, immune escape capability, and risk of reinfection as recently reviewed [3].

While the globe is still being ravaged by Delta VOC, a novel variant B.1.1.529, assigned as Omicron VOC, has surged in South Africa, and spread in dozens of countries so far, raising concern due to its potential association with increased transmissibility, reduction in vaccine effectiveness, and again increased risk of reinfections [4,5,6,7,8]. The Omicron has an odd mutational profile with an extremely high number of mutations, with 54 within the whole genome, particularly 34 within the Spike, and 6 in the Nucleocapsid. Remarkably, several Omicron VOC mutations are extremely rare in SARS-CoV-2 and have never defined any variant.

All VOCs, including Delta and Omicron, share some spike amino acid mutations in particular positions (such as K417N/T, L452R, E484K/A, T478K, N501Y) that can abrogate epitope recognition and thus may lead to evade both vaccine-induced immunity and innate immunity [3,9]. Notably, other mutations also alter the binding-affinity of specific drugs and have been potentially associated with hyper-susceptibility or resistance to drugs [10]. Finally, mutations, in particular deletions, could hamper a SARS-CoV-2 detection by diagnostic molecular assays [11]. In this light, the aim of our study is to describe the case of a patient infected with a Delta AY.4 strain characterized by a peculiar deletion in the Nucleocapsid, associated for the first-time with N gene target failure by the commercial Allplex^TM^ SARS-CoV2 Assay.

## 2. Materials and Methods

Total RNA was manually extracted from 280 µL of nasopharyngeal swab by using QIAamp viral RNA mini kit (Qiagen, Hilden, Germany) according to manufacturer’s instructions. Whole Genome Sequencing of SARS-CoV-2 RNA was performed using the library obtained according to COVIDSeq Assay Kit (Illumina Inc., San Diego, CA, USA). Finally, 15 pM of the denatured sample was sequenced by MiSeq Platform (Illumina Inc.) with MiSeq Reagent Kits v2 (2 × 150) (Illumina Inc., San Diego, CA, USA). Allplex^TM^ SARS-CoV2 Assay (Seegene Inc., Seoul, Korea), a multiplex real time PCR assay, was used to detect 4 target genes of SARS-CoV-2 (N, RdRp, S genes specific for SARS-CoV-2 and E gene for all Sarbecoviruses), and further detailed guidance can be found in the Supplementary Materials. FREND ™ COVID-19 Ag (NanoEnteK Inc., Seoul, Korea) is a fluorescence immunoassay (FIA) that was performed for qualitative detection of the SARS-CoV-2 N protein directly from nasopharyngeal swab specimens. The prevalence of N gene G214-G215 deletion in the most common Delta lineages and sublineages currently circulating in the globe and Europe were calculated based on Delta B.1.617.2. and AY.Xs sequences retrieved from GISAID by 12/01/2022 (AY.4 (N = 820703), AY.43 (N = 278501), B.1.617.2 (N = 144816), AY.4.2 (N = 83520), AY.98 (N = 41538), AY.122 (N = 197063), AY.4.2.2 (N = 27269), AY.23 (N = 18280), AY.103 (N = 278899), AY.121 (N = 33156), AY.39.1 (N = 24565), AY.39 (N = 49043), AY.99.2 (N = 20940), AY.25 (N = 130281), AY.44 (N = 238402), AY.3 (N = 146528), AY.119 (N = 34906), AY.126 (N = 38585)).

## 3. Results

Herein, we report a case of an Italian male infected by Delta sublineage AY.4 with the N G214-G215 deletion, leading to an N gene target failure (NGTF). The patient, aged 59 years, was unvaccinated and was admitted to the Respiratory Diseases Unit of the Tor Vergata Polyclinic for pulmonary embolism, with first negative results obtained by both Allplex^TM^ SARS-CoV2 Assay (Seegene) and rapid antigen test (FREND™ COVID-19 Ag). After several days of viral negativity, on 25 November 2021, he presented positive results for E and RdRP/S genes (cycle threshold [CT] of 21.04 and 22.32, respectively), but negative in N gene. In the following days, similar results were observed, with constantly negative N gene (Table 1). Interestingly, on 29 November, he also showed weakly positive results for the rapid SARS-CoV-2 N protein test (FREND™ COVID-19 Ag, NanoEnteK).

**Figure 1:**
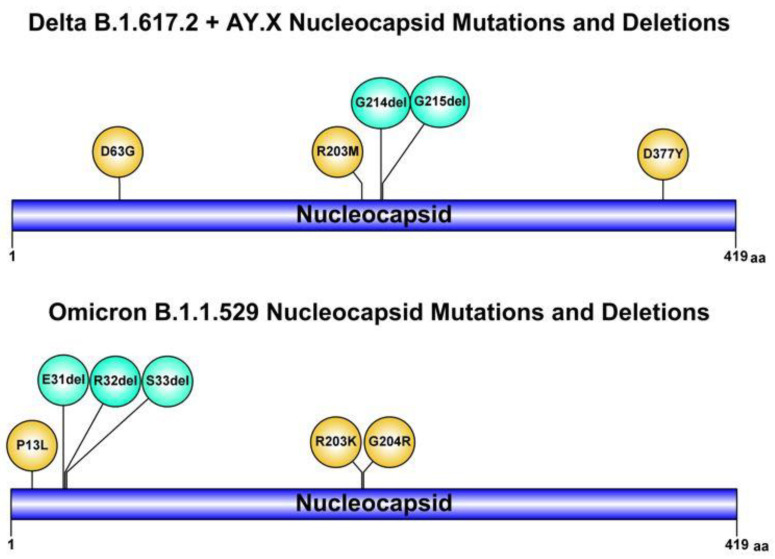
Schematic illustrations of mutations and deletions in SARS-CoV-2 Nucleocapsid of Delta and Omicron variants of concern, which may deceive diagnostic assays (created using graphical IBS 1.0.3 software).

Suspecting an infection with an atypical variant, and in particular the novel Omicron, the whole SARS-CoV-2 genome sequencing was rapidly performed by a nasopharyngeal swab sample. The sequence results (GISAID accession number: EPI_ISL_8712434) showed infection with the SARS-CoV-2 Delta sublineage AY.4 variant, characterized by an uncommon deletion of six nucleotides (28912_28917delTGGCGG, with an intra-patient prevalence of 100%) leading to G214-G215 deletion in the Nucleocapsid (Figure 1). So far, by analyzing over 250 SARS-CoV-2 sequences in our hospital, we could not identify someone with close contact/epidemiologically linked or infected with SARS-CoV-2 with this atypical deletion. The G214-G215 deletion is rarely occurring in most circulating Delta lineages and sub-lineages in Europe and particularly in Italy, with an overall prevalence never exceeding 0.2% (out of 2,618,373 Delta B.1.617.2.and AY.Xs sequences retrieved from GISAID by 12 January 2022). This deletion was linked with NGTF for the molecular Allplex^TM^ SARS-CoV2 Assay, as confirmed by consecutive N gene negativity. 

Likewise, this Delta sublineage AY.4 variant, the Omicron VOC also has an atypical deletion involving the positions 31-32-33 in the Nucleocapsid. The role of this deletion in favoring NGTF has not been clarified yet. Interestingly, the first case of Omicron VOC in Italy, diagnosed at the L. Sacco Hospital in Milan (GISAID accession number: EPI_ISL_6777160), showed positive results for all the four gene targets including N gene, using the same assay (Table 1).

## 4. Discussion

Herein, we report the first case, to our knowledge, of a SARS-CoV-2 Delta sublineage AY.4, with a specific rare G214-G215 deletion in the nucleocapsid, leading to an NGTF for the molecular Allplex^TM^ SARS-CoV2 Assay, but not to the rapid N antigen test (FREND™ COVID-19 Ag). By analyzing SARS-CoV-2 sequences from GISAID, the G214-G215 deletion, even if rarely detected, was found in 140 strains, mainly belonging to Delta lineages and sublineages (74.3%, 104/140). Interestingly, about 55% (77/140) of the strains are circulating in Europe and the remaining are sporadically dispersed in the rest of world. This deletion was reported for the first time in Scotland in late April 2020, and so far, it has an overall prevalence of 0.07% (N = 6.967.472, 11 January 2022 GISAID).

Conversely, positive results for all four gene targets, including the N gene, using the same assay, was found with the first case of Omicron VOC in Italy. Although the G214-G215 deletion in the N gene is not present in the Omicron VOC, this variant is characterized by two mutations (R023K and G204R) and a deletion of three amino acids (E31-R32-S33) that could lead to N gene detection failure in other assays specifically targeting this region. This point deserves further investigation.

Generally, specific mutations or deletions can localize in oligonucleotide primer/probe binding-sites used in real-time reverse-transcriptase polymerase chain reaction (rRT-PCR), potentially providing false-negative results to specific genes in some diagnostic assay. It is noteworthy to mention that the spike H69-V70 deletion of Alpha VOC was clearly associated with S gene target failure leading to partial false-negative results [11]. Interestingly, similar to Alpha VOC, Omicron has the known H69-V70 deletion in the Spike; hence, we can speculate that negativity to the spike target gene by assays specifically targeting this genomic region (as TaqPath test or other RT-PCR variant screening assays) can be used as an early marker in detection of Omicron VOC; a similar scenario occurred once Alpha VOC arose [11,12]. Moreover, some studies have reported cases related to NGTF. In particular, the mutation D3L, characterizing Alpha VOC, has been associated with N gene dropout and CT value shifting to Allplex™ SARS-CoV-2/FluA/FluB/RSV™ PCR assay [13]. Similarly, the single nucleotide polymorphism C927T in C terminal of the nucleocapsid has been reported to cause NGTF for an Xpert Xpress SARS-CoV-2 (GXP) assay [14,15], while being fully detected by the Allplex^TM^ SARS-CoV2 Assay [14]. 

Overall, this reinforces the need to use multiplex target PCR to bypass the lack of recognition of one gene target to not seriously affect SARS-CoV-2 diagnosis by providing possible false negative cases and re-evaluate the performance of the current assays by regularly optimizing the set of primers/probes used by molecular assays. All these aspects deserve further studies.

Finally, a limitation in this study is that the N gene target failure has been observed only by the assay currently used in our hospital for SARS-CoV-2 diagnosis. It is conceivable that other assays based on a different set of primers and probes could not provide the same results. In line with the abovementioned concept, this suggests the need to perform studies aimed at comparing the performance of the currently molecular assays for SARS-CoV-2 diagnosis according to viral genetic variability.

## 5. Conclusions

Our results emphasize the importance to perform whole genome SARS-CoV-2 sequencing and to characterize novel mutations and/or deletions that could jeopardize the proper interpretation of diagnostic tests.

## Figures and Tables

**Table 1:** Patients’ characteristics at different time-points with their relevant results.

Patient	Diagnostic Date	VOC	Test * Date	N Gene CT	RdRP/S Gene CT	E Gene CT
**Case P1**	25 November 2021	Delta AY.4	25 November 2021	**Negative**	22.32	21.04
			26 November 2021	**Negative**	28.97	26.50
			29 November 2021	**Negative**	24.14	23.51
**Case P2**	16 November 2021	Omicron	26 November 2021	20.28	18.91	18.07

* Tested using Allplex^TM^ SARS-CoV2 Assay (Seegene). Abbreviations: VOC, variant of concern; CT, cycle threshold.

## Data Availability

GISAID accession number: EPI_ISL_8712434 and EPI_ISL_6777160 https://www.gisaid.org/ (accessed on 12 December 2021).

## Supplementary Materials

The following supporting information can be downloaded at: https://www.mdpi.com/article/10.3390/microorganisms10020268/s1, file title: Seegene Allplex^TM^ SARS-CoV-2 Guidelines.
