# Supplementary material for: First Case of a COVID-19 Patient Infected by Delta AY.4 with a Rare Deletion Leading to a N Gene Target Failure by a Specific Real Time PCR Assay: Novel Omicron VOC Might Be Doing Similar Scenario?
Source: Microorganisms. 2022 Jan 25;10(2):268. doi: 10.3390/microorganisms10020268 (PMC8875198; doi:10.3390/microorganisms10020268)
Supplement: Supplementary file 1 [file microorganisms-10-00268-s001.zip › microorganisms-1534145-supplementary.pdf]

For Professional Use Only

Allplex™

SARS-CoV-2 Assay

(Cat.No. RV10248X)

Multiplex real-time one-step RT-PCR system for detection of SARS-CoV-2 from nasopharyngeal aspirate, nasopharyngeal swab, bronchoalveolar lavage, oropharyngeal (throat) swab, and sputum.

For use with

1. Microlab NIMBUS IVD and Microlab STARlet IVD

2. Seegene NIMBUS and Seegene STARlet

For use with

1. CFX96™ Real-time PCR System (CFX Manager™ Software-IVD v1.6)

2. CFX96™ Dx System (CFX Manager™ Dx Software v3.1)

3. Applied Biosystems™ 7500 (SDS software v2.0.5)

100

IVD **For *in vitro* diagnostic use only**

Seegene Inc.,
Taewon Bldg., 91 Ogeum-ro, Songpa-gu, Seoul, Republic of Korea 05548

EC REP **Medical Technology Promedt Consulting GmbH**
Altenhofstrasse 80, D-66386 St.Ingbert, Germany

Not available in the U.S.

TABLE OF CONTENTS

NOTICES ---------- 3

INTENDED USE ---------- 4

PRINCIPLES AND PROCEDURE OVERVIEW ---------- 5

BACKGROUND INFORMATION ---------- 6

REAGENTS ---------- 7

STORAGE AND HANDLING ---------- 8

MATERIALS REQUIRED BUT NOT PROVIDED ---------- 8

PROTOCOL ---------- 9

REAL-TIME PCR INSTRUMENT SET UP AND RESULTS ANALYSIS ---------- 17

- CFX96™ Real-time PCR System (CFX Manager™ Software-IVD v1.6) ------ 17
- CFX96™ Dx System (CFX Manager™ Dx Software v3.1) ---------- 27
- Applied Biosystems™ 7500 (SDS software v2.0.5) ---------- 37

RESULTS ---------- 46

TROUBLESHOOTING ---------- 50

PERFORMANCE ---------- 52

REFERENCES ---------- 57

SYMBOLS ---------- 59

ORDERING INFORMATION ---------- 60

NOTICES

- For *in vitro* diagnostic use only.
- The Allplex™ SARS-CoV-2 Assay should be performed by qualified, trained personnel.
- Reliability of the results depends on adequate specimen collection, storage, transport, and processing procedure.
- **This product is only for use with Microlab NIMBUS IVD, Microlab STARlet IVD, Seegene NIMBUS, and Seegene STARlet in maximum 5 separate runs.**
- **This test has been validated for the following specimen types: nasopharyngeal aspirate, nasopharyngeal swab, bronchoalveolar lavage, oropharyngeal (throat) swab, and sputum.** This test has not been validated for any other types of specimens.
- **Store RNA samples at ≤ -20°C until use and keep on ice during use.**
- Sensitivity of the assay may decrease if samples are repeatedly frozen/thawed or stored for a longer period of time.
- Workflow in a laboratory should proceed in an unidirectional manner.
- Wear disposable gloves and change them before entering different areas. Change gloves immediately if contaminated or treat them with DNA decontaminating reagent.
- Supplies and equipment must be dedicated to working areas and should not be moved from one area to another.
- Do not pipette by mouth.
- Do not eat, drink or smoke in laboratory work areas. Wear disposable powder-free gloves, laboratory coats and eye protections when handling specimens and reagents. Wash hands thoroughly after handling specimens and test reagents.
- Avoid contamination of reagents when removing aliquots from reagent tubes. Use of sterilized aerosol resistant disposable pipette tips is recommended.
- Do not pool reagents from different lots or from different tubes of the same lot.
- Do not use the product after its expiry date.
- Do not reuse all disposable items.
- Use screw-capped tubes and prevent any potential splashing or cross-contamination of specimens during preparation.
- Be careful not to contaminate reagents with extracted nucleic acids, PCR products, and positive controls. To prevent contamination of reagents, the use of filter-tips is recommended.
- Use separated and segregated working areas for each experiment.
- To avoid contamination of working areas with amplified products, open PCR reaction tubes

or strips only at designated working areas after amplification.

- Store positive materials separately from the kit's reagents.
- Laboratory safety procedures (refer to Biosafety in Microbiological and Biomedical Laboratories & CLSI Documents) must be taken when handling specimens. Thoroughly clean and disinfect all work surfaces with 0.5% sodium hypochlorite (in de-ionized or distilled water). Product components including product residuals and packaging can be considered as laboratory waste. Dispose of unused reagents and waste in accordance with applicable federal, state, and local regulations.
- Expiry date is 13 months from the date of manufacture at ≤ -20°C. Please refer to label for the expiry date.
- Clinical correlation with patient history and other diagnostic information is necessary to determine patient infection status.
- Seegene NIMBUS and Seegene STARlet are the same equipment as the Microlab NIMBUS IVD and Microlab STARlet IVD, respectively although the manufacturers are different from each other. Since there are no hardware changes on the devices, the test results are the same for those.
- The brand name of "CFX96™ Real-time PCR Detection System-IVD" has been changed to "CFX96™ Dx system". Since there are no hardware changes on the systems, it will be expected to obtain the same results from both systems.
- "CFX Manager™ Dx Software v3.1" is an upgrade version of "CFX Manager™ Software-IVD v1.6". The upgraded software includes enhancements to the "Run" menu. These enhancements do not impact the results of data analysis; therefore, the results will be the same.
- This kit is a qualitative *in vitro* test for the single or multiple detection of 4 types of gene (E gene, RdRP gene, S gene, and N gene).

INTENDED USE

Allplex™ SARS-CoV-2 Assay is *in vitro* diagnostic medical device designed for qualitative detection of SARS-CoV-2 with real-time reverse transcription PCR from nasopharyngeal aspirate, nasopharyngeal swab, bronchoalveolar lavage, oropharyngeal (throat) swab, and sputum.

PRINCIPLES AND PROCEDURE OVERVIEW

1. Principles

Allplex™ SARS-CoV-2 Assay is a multiplex real-time RT-PCR assay that enables simultaneous amplification and detection of target nucleic acids of E gene, RdRP gene, S gene, and N gene with Internal Control (IC).The presence of specific gene sequences in the reaction is reported as a Ct value through Seegene Viewer analysis software.

An exogenous gene is used as Internal Control (IC) to monitor the whole process of nucleic acid extraction and to check for any possible PCR inhibition.

To prevent amplification product from acting as potential contaminants, Uracil-DNA glycosylase (UDG)-dUTP system is employed in Allplex™ SARS-CoV-2 Assay. The UDG-dUTP system is commonly used when performing PCR to eliminate amplicon carry-over using UDG to excise uracil residues from DNA by cleaving the N-glycosylic bond.

2. Procedure Overview

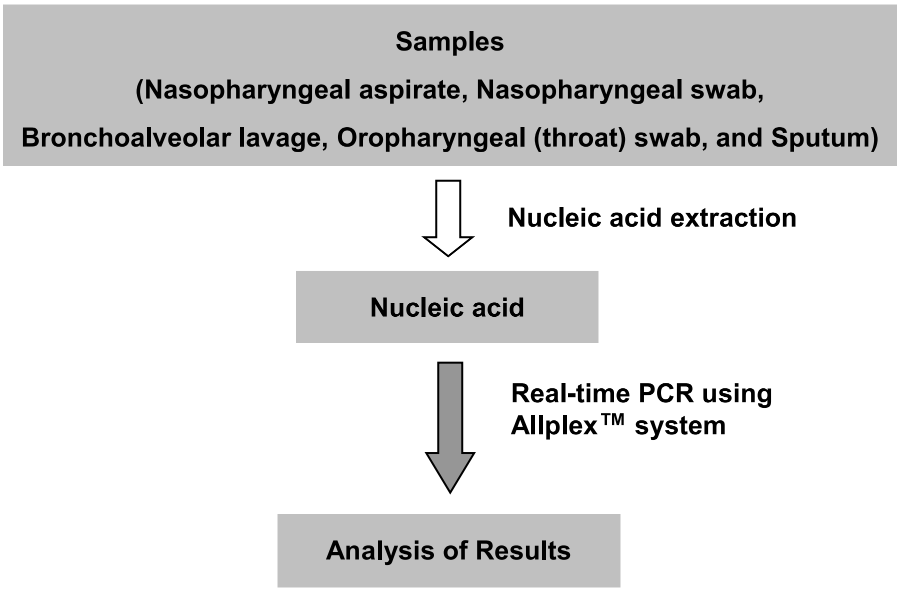

< Allplex™ SARS-CoV-2 Assay procedure overview >

BACKGROUND INFORMATION

1. Severe acute respiratory syndrome coronavirus 2 (SARS-CoV-2)

Severe acute respiratory syndrome coronavirus 2 (SARS-CoV-2), previously known by the provisional name 2019 novel coronavirus (2019-nCoV), is the cause of the respiratory coronavirus disease 2019 (COVID-19). Taxonomically, it is a strain of the Severe acute respiratory syndrome-related coronavirus (SARSr-CoV), a positive-sense single-stranded RNA virus. It is contagious in humans, and the World Health Organization (WHO) has designated the ongoing pandemic of COVID-19 a Public Health Emergency of International Concern.

SARS-CoV-2 is believed to have zoonotic origins. It has close genetic similarity to bat coronaviruses, suggesting it emerged from a bat-borne virus. An intermediate animal reservoir such as a pangolin is also thought to be involved in its introduction to humans. Chinese scientists first isolated SARS-CoV-2 7 January 2020 from patients in Wuhan, China who had had pneumonia of unknown cause in December 2019.

REAGENTS

The reagents contained in one kit are sufficient for 100 reactions.

Order information (REF Cat. No. RV10248X)

Allplex™ SARS-CoV-2 Assay			
**Symbol**	**Contents**	**Volume**	**Description**
PRIMER	SARS2 MOM	500 μL	Oligo Mix :<br>- Amplification and detection reagent
PREMIX	EM8	500 μL	- RTase<br>- DNA polymerase<br>- Uracil-DNA glycosylase (UDG)<br>- Buffer containing dNTPs
CONTROL +	SARS2 PC	50 μL	Positive Control (PC):<br>- Mixture of pathogen and IC clones
CONTROL IC	RP-V IC 2	1,000 μL	Exogenous Internal Control (IC)
WATER	RNase-free Water	1,000 μL	Ultrapure quality, PCR-grade
	User manual		

STORAGE AND HANDLING

All components of Allplex™ SARS-CoV-2 Assay should be stored at ≤ -20°C. All components are stable under recommended storage conditions until the expiry date stated on the label. The performance of kit components is not affected for up to 5 freezing and thawing. If the reagents are to be used only intermittently, they should be stored in aliquots.

MATERIALS REQUIRED BUT NOT PROVIDED

- Disposable powder free gloves (latex or nitrile)
- Pipettes (adjustable) and Sterile pipette tips
- 1.5 mL microcentrifuge tubes
- Nucleic acid extraction kit (see Nucleic Acid Extraction)
- Clean bench
- Ice Maker
- Desktop centrifuge
- Vortex mixer
- CFX96™ Real-time PCR Detection system (Bio-Rad)
- CFX96™ Dx System (Bio-Rad)
- Applied Biosystems™ 7500 (Thermo Fisher Scientific)
- Low-Profile 0.2 mL 8-Tube Strips without Caps (white color, Cat. No. TLS0851, Bio-Rad)*
- Optical Flat 8-Cap Strips (Cat. No. TCS0803, Bio-Rad)*
- Hard-Shell® 96-Well PCR Plates, low profile, thin wall, skirted, white/white (Cat. No. HSP9655, Bio-Rad)*
- Hard-Shell® 96-Well PCR Plates, low profile, thin wall, skirted, white/white, barcoded (Cat. No. HSP9955, Bio-Rad)*
- Permanent Clear Heat Seal (Cat. No. 1814035, Bio-Rad)* §
- PX1 PCR plate sealer (auto-sealer, Cat. No. 181-4000, Bio-Rad)* §
- EU 0.1ml 8-tube strip, LP, W, Extra Robust (Cat. No. B72719, BIOplastics)*
- EU Optical Wide area 8-Cap Strip (Cat. No. B57801, BIOplastics)*
- 96 x 0.1ml Plate, LP, W, FULL, 96 well plate (Cat. No. B70679, BIOplastics)*
- Opti-Seal Optical Sealing Sheet (Cat. No. 157300, BIOplastics)*
- MicroAmp® Optical 96-Well Reaction Plate (Cat. No. N8010560, Thermo Fisher Scientific)**
- MicroAmp™ Optical 96-Well Reaction Plate with Barcode (Cat. No. 4306737, Thermo Fisher Scientific)**
- Optical Adhesive Covers (Cat No. 4360954, Thermo Fisher Scientific)**
- MicroAmp™ Optical 8-Tube Strip, 0.2 mL (Cat No. 4316567, Thermo Fisher Scientific)**
- MicroAmp™ Optical 8-Cap Strips (Cat No. 4323032, Thermo Fisher Scientific)**

* Products for CFX96™ Real-time PCR Detection and CFX96™ Dx System
** Products for Applied Biosystems™ 7500
§ Make sure to use the heat seal and the plate sealer listed above together.

PROTOCOL

1. Specimen Collection, Storage, and Transport

Note: All samples should be treated as potentially infectious materials. Only permitted are those sample materials, which are collected, transported and stored by attending strictly to the following rules and instructions.
Note: To ensure high quality of samples, samples should be transported as fast as possible at indicated temperature.

A. Specimen Collection

Nasopharyngeal aspirate, Nasopharyngeal swab, Bronchoalveolar lavage, Oropharyngeal (throat) swab

- Nasopharyngeal aspirate, nasopharyngeal swab, bronchoalveolar lavage, oropharyngeal (throat) swab specimens are routinely examined for common respiratory pathogens.
- Obtaining respiratory specimens may be difficult in some patients. In such cases, nasopharyngeal swabs may be collected simply and efficiently using new nylon flocked swabs (COPAN, Italy) and Universal Transport Medium (UTM).

Sputum

- Give clear instructions to patients when collecting sputum specimens. Patients must either collect samples outside in the open air or away from other people. Patients should not collect samples in confined spaces such as toilets.
- Rinse mouth with water before collecting sputum. The patient should cough deeply and expectorate sputum directly into the container.
- A sputum sample must have a volume of 3~5 mL.

Manufacturer	Specimen collection device	Cat. No.
COPAN	ESwab	482CE
COPAN	ENAT PM 2ML PERNASAL APPLICATOR**	606CS01P*
COPAN	UTM with Flocked Swabs	360C / 305C
DIAGNOSTIC HYBRIDS	UTM with Flexible Minitip Flocked Swab	403C / 406C
COPAN	MSwab® kit	6E012N / 6E013N
COPAN	MSwab® bulk	6E011N
Noble Biosciences	CTM (Clinical Virus Transport Medium)	UTNFS-3B-2-N1P / UTNFS-3B-2

SG Medical	GeneTM Set (GTS2)**	T5001
SG Medical	GeneTM Set (GTS1)**	T5002

*Please use catalog numbers shown above to purchase products from Seegene Inc.

** ENAT PM 2ML PERNASAL APPLICATOR, GeneTM Set (GTS2) and GeneTM Set (GTS1) are not applicable to extraction-free method.

B. Specimen Storage & Transport

Specimen	Storage & Transport		Note
	Temp.	Duration*	
Nasopharyngeal aspirate	2~8°C	3 days	- Performance may be affected by prolonged storage of specimens.<br>- Specimens should also adhere to local and national instructions for transport of pathogenic materials.
Nasopharyngeal swab			
Bronchoalveolar lavage			
Oropharyngeal (throat) swab			
Sputum			

* Duration: The time period from the specimen collection to the final test (includes transport and storage of specimens prior to tests).

2. Nucleic Acid Extraction

[Extraction methods in different specimens]

Note: Please use the automated extraction system according to the specimen shown in the following table.

Specimen	Automated Extraction System							Extraction-free***§
	Microlab NIMBUS IVD / STARlet IVD		Seegene NIMBUS / STARlet		KingFisher Flex*	MagNA Pure 96*	Maelstrom™ 9600**	
	Universal Cartridge Kit	Viral DNA/RNA 200 C Kit ***	Universal Cartridge Kit	Viral DNA/RNA 200 C Kit ***				
Nasopharyngeal aspirate	O	X	O	X	O	O	X	X
Nasopharyngeal swab	O	O	O	O	O	O	O	O
Bronchoalveolar lavage	O	X	O	X	X	X	X	X
Oropharyngeal (throat) swab	O	O	O	O	O	O	O	O
Sputum	O	X	O	X	O	O	O	X

* Bronchoalveolar lavage has not been validated with KingFisher Flex and MagNA Pure 96.
** Nasopharyngeal aspirate and bronchoalveolar lavage have not been validated with Maelstrom™ 9600.
*** Nasopharyngeal aspirate, bronchoalveolar lavage and sputum have not been validated with STARMag 96 X 4 Viral DNA/RNA 200 C Kit and extraction-free method.
§ Swab specimens using ENAT PM 2ML PERNASAL APPLICATOR, GeneTM Set (GTS2) and GeneTM Set (GTS1) are not applicable to extraction-free method.

2-1. Standard Extraction

A. Pre-treatment of specimen

Sputum

- Add 2 volumes of 1X PBS or saline solution to the 1 volume specimen in the 15 mL conical tube and vortex thoroughly to disperse the sample.
- Transfer recommend volume (See Recommended Vol. of 2-C) of sample to a new tube.
- Follow the extraction kit protocol.

Note: In case of the specimens without viscosity, pre- treatment step is NOT required.

B. Internal Control

Note: IC, included in the kit, allows the user not only to verify the nucleic acid extraction procedure, but also to identify any PCR inhibition.

- RP-V IC 2 tube must be loaded on Microlab NIMBUS IVD, Microlab STARlet IVD, Seegene NIMBUS or Seegene STARlet before nucleic acid extraction.
- When using KingFisher Flex, MagNA Pure 96 or Maelstrom™ 9600, 10 μL of RP-V IC 2 must be added to each specimen before nucleic acid extraction.

C. Automated Nucleic Acid Extraction System

Note: Please use the recommended volumes of specimen and elution as indicated below. For other matters, refer to the manufacturer's manual.

C-1. Microlab NIMBUS IVD

Note: See **Microlab NIMBUS IVD** operation manual.

Automated Extraction System	Manufacturer	Cat. No.	Recommended Vol.
Microlab NIMBUS IVD	Hamilton	65415-02*	-
STARMag 96 X 4 Universal Cartridge Kit	Seegene	744300.4.<br>UC384	Specimen: 300 μL<br>Elution: 100 μL
STARMag 96 X 4 Viral DNA/RNA<br>200 C Kit**	Seegene	EX00013C	Specimen: 300 μL<br>Elution: 100 μL

* Please use catalog numbers shown above to purchase products from Seegene Inc.
** Nasopharyngeal aspirate, bronchoalveolar lavage, and sputum have not been validated.

C-2. Microlab STARlet IVD

Note: See **Microlab STARlet IVD** operation manual.

Automated Extraction System	Manufacturer	Cat. No.	Recommended Vol.
Microlab STARlet IVD	Hamilton	173000-075*	-
STARMag 96 X 4 Universal Cartridge Kit	Seegene	744300.4.<br>UC384	Specimen: 300 μL<br>Elution: 100 μL
STARMag 96 X 4 Viral DNA/RNA<br>200 C Kit**	Seegene	EX00013C	Specimen: 300 μL<br>Elution: 100 μL

* Please use catalog numbers shown above to purchase products from Seegene Inc.
** Nasopharyngeal aspirate, bronchoalveolar lavage, and sputum have not been validated.

C-3. Seegene NIMBUS

Note: See **Seegene NIMBUS** operation manual.

Automated Extraction System	Manufacturer	Cat. No.	Recommended Vol.
Seegene NIMBUS	Seegene	65415-03	-
STARMag 96 X 4 Universal Cartridge Kit	Seegene	744300.4.<br>UC384	Specimen: 300 μL<br>Elution: 100 μL
STARMag 96 X 4 Viral DNA/RNA 200 C Kit*	Seegene	EX00013C	Specimen: 300 μL<br>Elution: 100 μL

* Nasopharyngeal aspirate, bronchoalveolar lavage, and sputum have not been validated.

C-4. Seegene STARlet

Note: See **Seegene STARlet** operation manual.

Automated Extraction System	Manufacturer	Cat. No.	Recommended Vol.
Seegene STARlet	Seegene	67930-03	-
STARMag 96 X 4 Universal Cartridge Kit	Seegene	744300.4.<br>UC384	Specimen: 300 μL<br>Elution: 100 μL
STARMag 96 X 4 Viral DNA/RNA 200 C Kit*	Seegene	EX00013C	Specimen: 300 μL<br>Elution: 100 μL

* Nasopharyngeal aspirate, bronchoalveolar lavage, and sputum have not been validated.

C-5. KingFisher™ Flex Purification System, KingFisher with 96 Deep-well Head

Note: See **KingFisher Flex** operation manual.

Automated Extraction System	Manufacturer	Cat. No.	Recommended Vol.
KingFisher™ Flex Purification System, KingFisher with 96 Deep-well Head	Thermo Fisher Scientific	5400630	-
MagMAX™ Viral/Pathogen Nucleic Acid Isolation Kit	Thermo Fisher Scientific	A42352	Specimen: 200 μL<br>Elution: 80 μL

Bronchoalveolar lavage has not been validated with KingFisher Flex.

C-6. MagNA Pure 96

Note: See **MagNA Pure 96** operation manual.

Automated Extraction System	Manufacturer	Cat. No.	Recommended Vol.
MagNA Pure 96	Roche Diagnostics	06541089001	-
MagNA Pure 96 DNA and Viral NA Small Volume Kit	Roche Diagnostics	06543588001	Specimen: 200 μL<br>Elution: 100 μL

Bronchoalveolar lavage has not been validated with MagNA Pure 96.

C-7. Maelstrom™ 9600

- Proceed the extraction process using **'<u>665-Rapid</u>' protocol**

Automated Extraction System	Manufacturer	Cat. No.	Recommended Vol.
Maelstrom™ 9600	Taiwan Advanced Nanotech Inc.	M9600	-
TANBead® Nucleic Acid Extraction Kit OptiPure Viral Auto Tube	Taiwan Advanced Nanotech Inc.	W665S66	Specimen: 300 μL<br>Elution: 80 μL
TANBead® Nucleic Acid Extraction Kit OptiPure Viral Auto Plate	Taiwan Advanced Nanotech Inc.	W665A46	Specimen: 300 μL<br>Elution: 80 μL
TANBead® Nucleic Acid Extraction Kit OptiPure Viral Bulk Plate	Taiwan Advanced Nanotech Inc.	W665A10	Specimen: 300 μL<br>Elution: 80 μL

Nasopharyngeal aspirate and bronchoalveolar lavage have not been validated with Maelstrom™ 9600.

2-2. Extraction-free Method

Note: RP-V IC 2 must be added to Mastermix during preparation for PCR. See *Preparation for Real-time One-step RT-PCR* (page 14) for more information.

1) Aliquot 45 μL of Nuclease-free water into PCR tubes
2) Add 15 μL of each sample into the tube containing aliquot of the Nuclease-free water
3) Close the cap, quick vortex and briefly centrifuge
4) Incubate at 98℃ for 3 min*
5) Chill at 4℃ for 5 min*

*Note: It is recommended to perform steps 4)~5) on PCR instrument with PCR tube attached individual caps.

Note: If you need retesting, start with the original sample not the lysate.

3. Preparation for Real-time One-step RT-PCR

Note: Correct tubes and caps must be used (see MATERIALS REQUIRED BUT NOT PROVIDED).

Note: Aerosol resistant filter tips and tight gloves must be used when preparing One-step RT-PCR reactions. Use extreme care to prevent cross-contamination.

Note: Completely thaw all reagents on ice.

Note: Briefly centrifuge reagent tubes to collect residual drops inside of the cap.

Note: The steps A~D are automatically processed on Microlab NIMBUS IVD, Microlab STARlet IVD, Seegene NIMBUS and Seegene STARlet. Refer to each operation manual.

A. Prepare the Reaction Mastermix

A-1 Standard extraction

5 μL	SARS2 MOM
5 μL	EM8
5 μL	RNase-free Water
15 μL	Total volume of Mastermix

A-2 Extraction-free method

5 μL	SARS2 MOM
5 μL	EM8
4 μL	RNase-free Water
1 μL	RP-V IC 2
15 μL	Total volume of Mastermix

Note: Calculate the total amount of each reagent needed based on the number of reactions including samples and controls.

B. Mix by quick vortexing, and briefly centrifuge.

C. Aliquot 15 μL of Reaction Mastermix into PCR tubes.

D. Add 5 μL of each sample's nucleic acids into the tube containing Reaction Mastermix.

15 μL	Reaction Mastermix
5 μL	Sample's nucleic acid
20 μL	Total volume of reaction

E. Close and briefly centrifuge the PCR tubes.

F. Verify that the liquid containing all PCR components is at the bottom of each PCR tube. If not, centrifuge again at a higher rpm for a longer time.

Note: It is recommended to centrifuge PCR tubes before PCR to eliminate air bubbles and collect all residual liquids at the bottom of tubes.

Correct	Incorrect
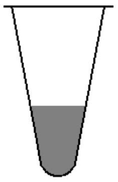	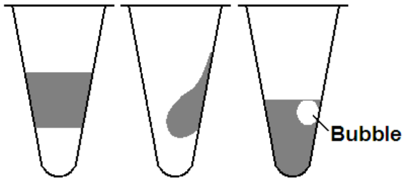

Note: Use a new sterile pipette tip for each sample.

Note: For **Negative Control (NC)**, use 5 μL of "**RNase-free Water**" instead of sample's nucleic acid.

Note: For **Positive Control (PC)**, use 5 μL of "**SARS2 PC**" instead of sample's nucleic acid.

Note: Be careful not to cross-contaminate the Reaction Mastermix and samples with the Positive Control.

Note: Do not label the reaction tube on its cap. Fluorescence is detected from the top of each reaction tube.

REAL-TIME PCR INSTRUMENT SET UP AND RESULT ANALYSIS

1. CFX96™ Real-time PCR Detection System (CFX Manager™ Software-IVD v1.6)

1.1. Real-time PCR Instrument Setup

Note: CFX96™ Real-time PCR Detection System (Bio-Rad) experiment setup can be divided into three steps: Protocol Setup, Plate Setup, and Start run.

A. Protocol Setup

1) In the main menu, select **"File"** → **"New"** → **"Protocol"** to open **"Protocol Editor"**.

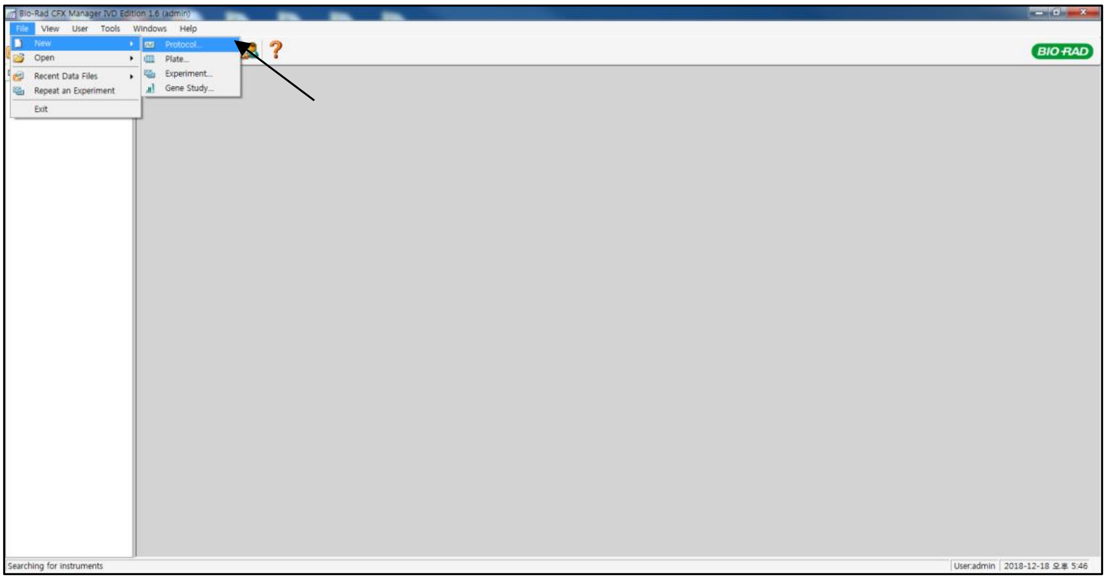

Fig. 1. **Protocol Setup**

2) In **"Protocol Editor"**, define the thermal profile as follows:

Step	No. of cycles	Temperature	Duration
1	1	50°C	20 min
2		95°C	15 min
3	45	95°C	10 sec
4*		60°C	15 sec
5*		72°C	10 sec
6	GOTO Step 3, 44 more times		

Note*: Plate Read Step. Fluorescence is detected at 60°C and 72°C.

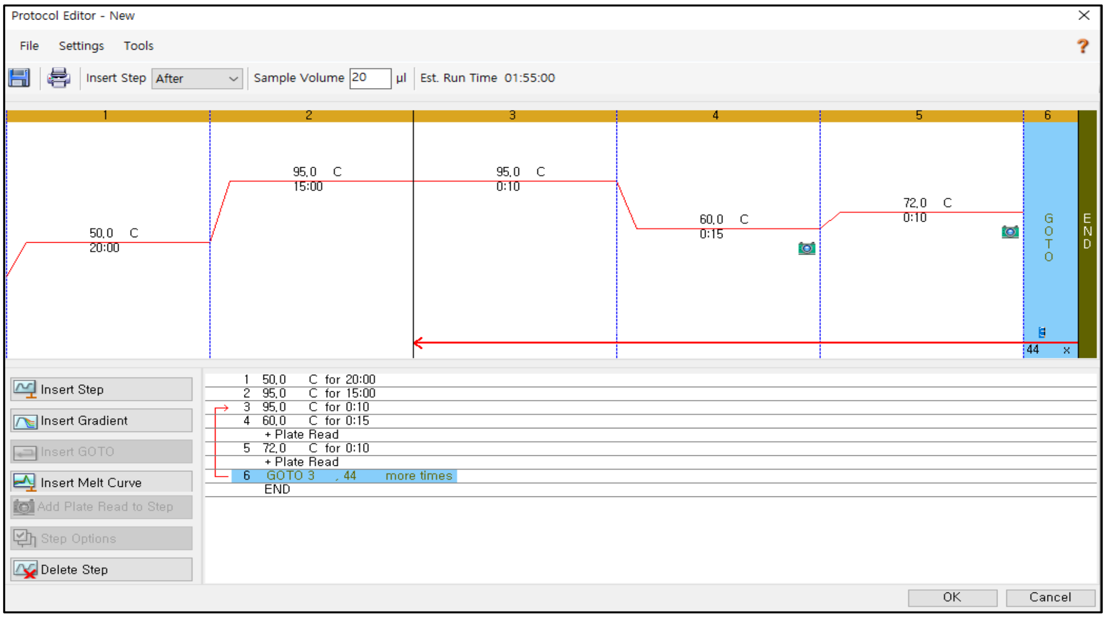

Fig. 2. **Protocol Editor**

3) Click the box next to **"Sample Volume"** to directly input 20 µL.

4) Click **"OK"** and save the protocol to open the **"Experiment Setup"** window.

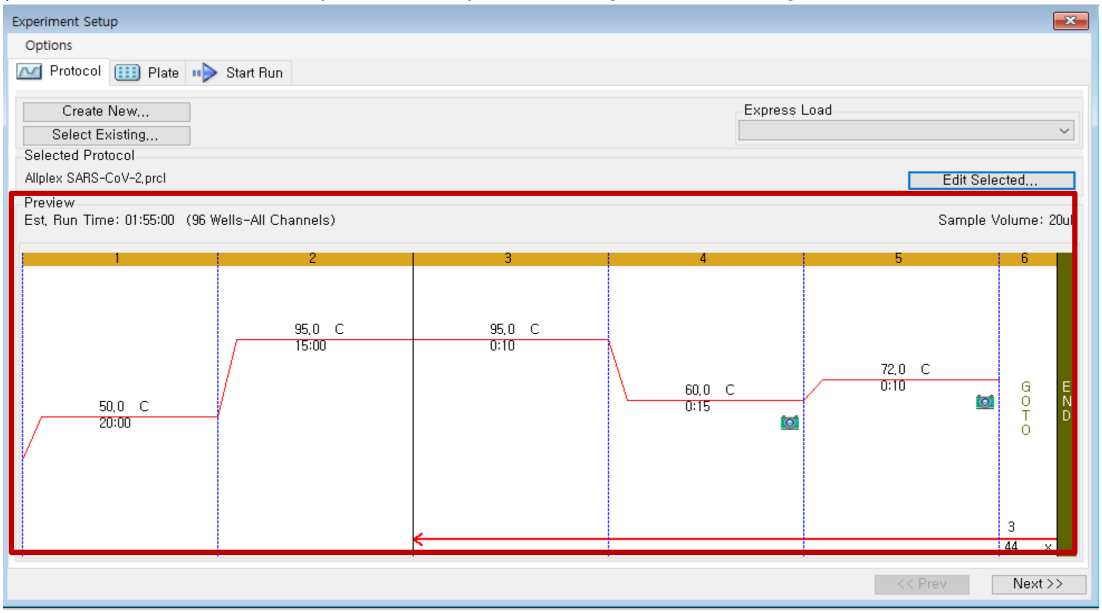

Fig. 3. **Experiment Setup: Protocol**

B. Plate Setup

1) From **"Plate"** tab in **"Experiment Setup"**, click **"Create New"** to open **"Plate Editor"** window.

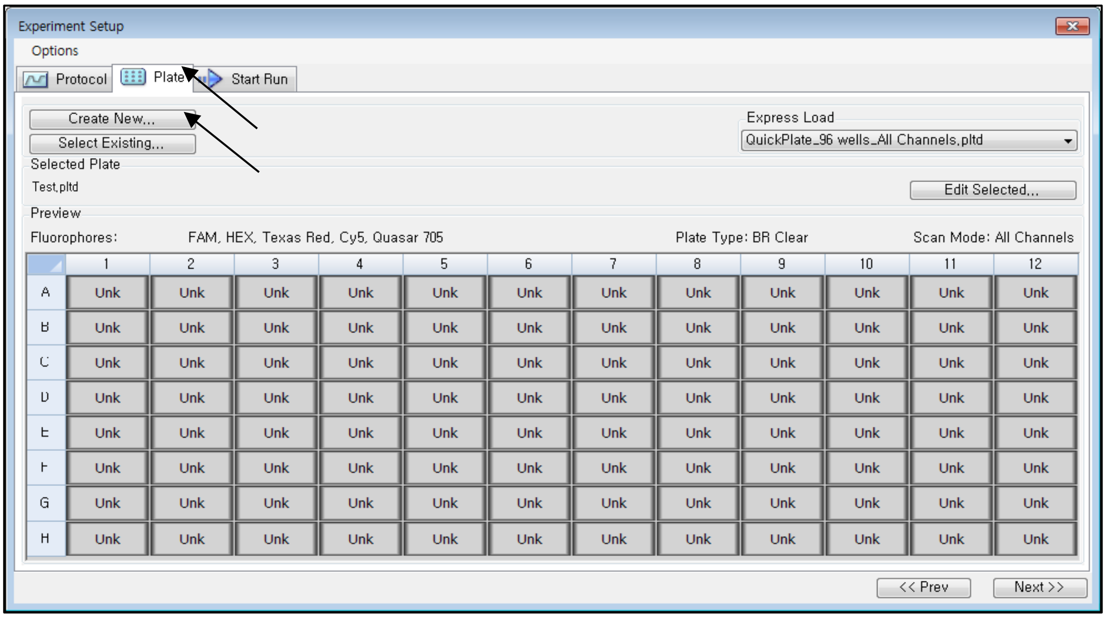

Fig. 4. **Plate Editor**

2) Click **“Select Fluorophores”** to indicate the fluorophores (***FAM***, ***HEX***, ***Cal Red 610***, ***Quasar 670***) that will be used and click **“OK”**.

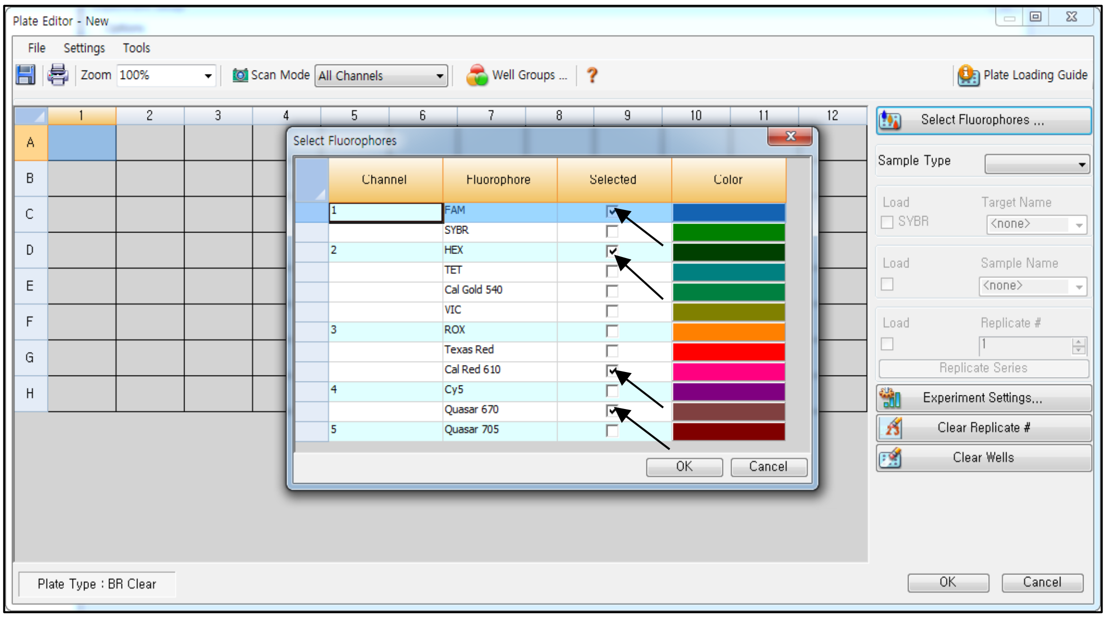

Fig. 5. **Select Fluorophores** (***FAM***, ***HEX***, ***Cal Red 610***, *and* ***Quasar 670***)

3) Select the wells where the PCR tube will be placed and select their sample types from the **“Sample Type”** drop-down menu.

- ***Unknown***: *Clinical samples*
- ***Negative Control***
- ***Positive Control***

4) Click on the appropriate checkboxes (***FAM, HEX, Cal Red 610, and Quasar 670***) to specify the fluorophores to be detected in the selected wells.

5) Type **“Sample Name”** and press enter key.

6) In **“Settings”** of the **“Plate Editor”** main menu, choose the **“Plate Size”** (***96 wells***) and **“Plate Type”** (***BR White***).

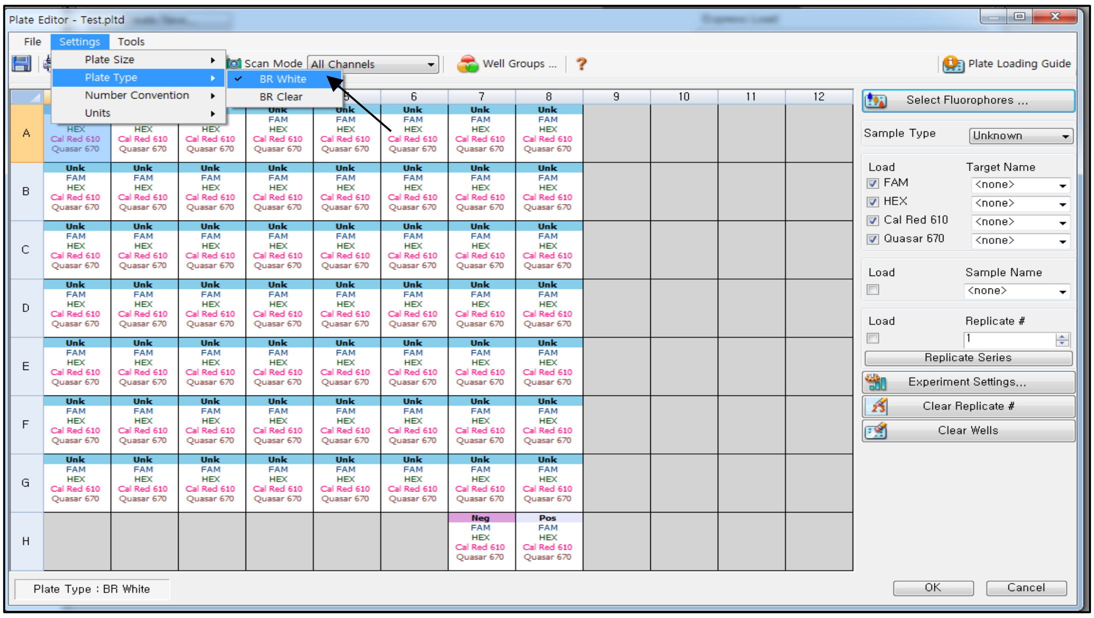

Fig. 6. **Plate Setup**

7) Click **“OK”** to save the new plate.

8) You will be returned to the **“Experiment Setup”** window.

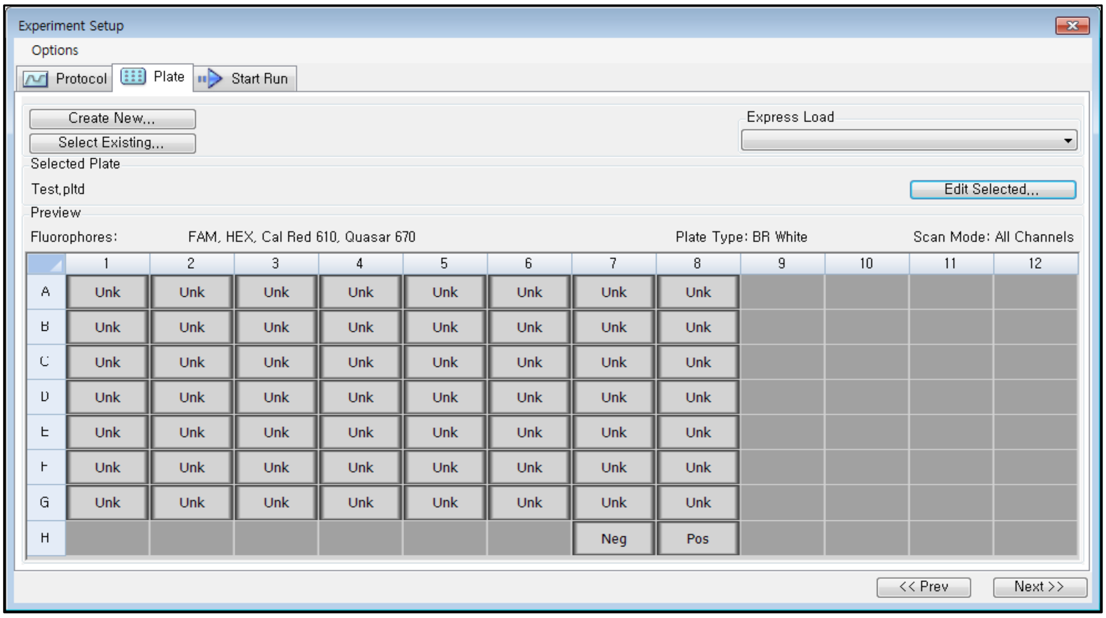

Fig. 7. **Experiment Setup: Plate**

9) Click **“Next”** to Start Run.

C. Start Run

1) From **"Start Run"** tab in **"Experiment Setup"**, click **"Close Lid"** to close the instrument lid.

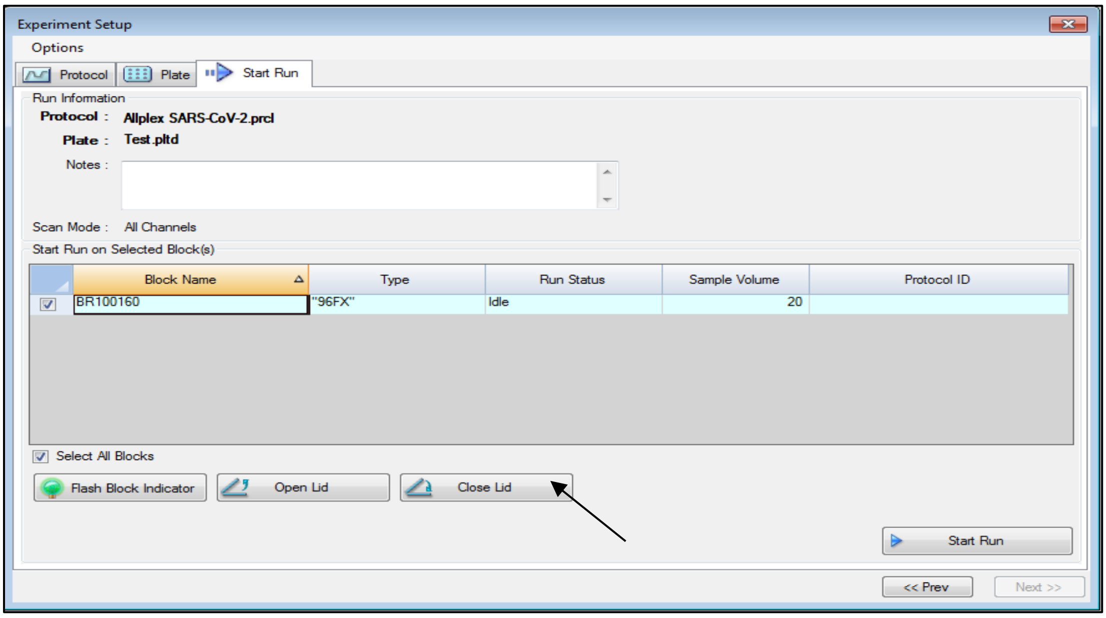

Fig. 8. **"Close Lid"**

2) Click **"Start Run"**.

3) Store the run file either in My Documents or in a designated folder. Input the file name, click **"SAVE"**, and the run will start.

1.2. Data Analysis

A. Create folders for data export

1) To save data of all detection steps of amplification curves from the result file, create one folder.

2) Folder name may be as desired by user (For 'Seegene Export' function, folders "QuantStep4" and "QuantStep5" are automatically created to save each amplification curve data under the folder created by user).

B. Pre-settings for Data Analysis in CFX96™

1) After the test, click the **"Quantitation"** tab to see the amplification curve results.

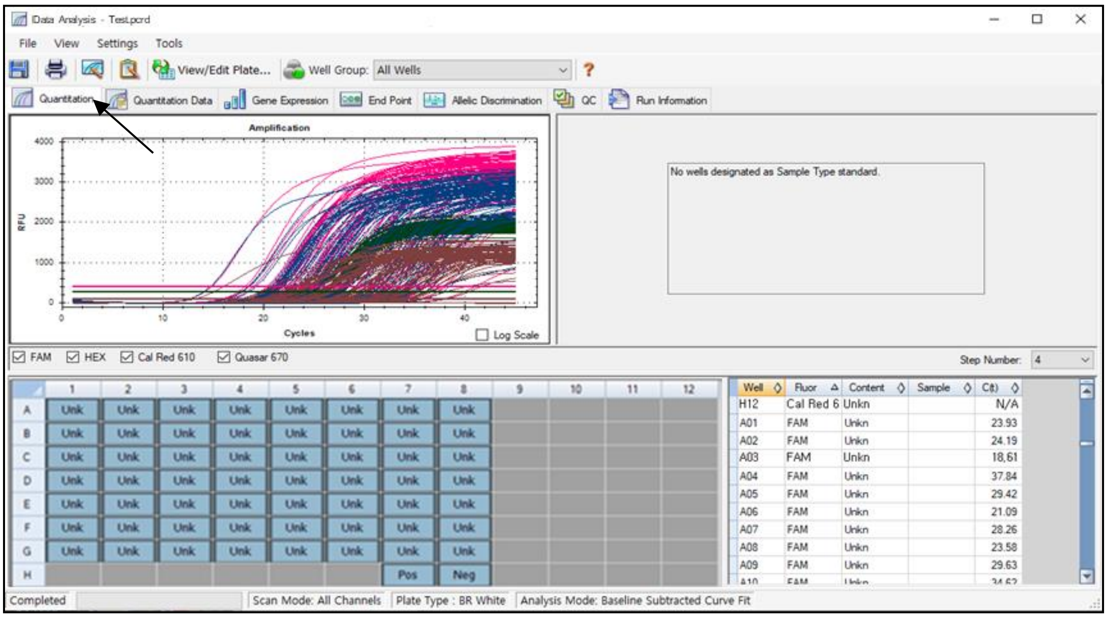

Fig. 9. **Amplification curve results**

2) Select **"No Baseline Subtraction"** from Analysis Mode of Settings menu.

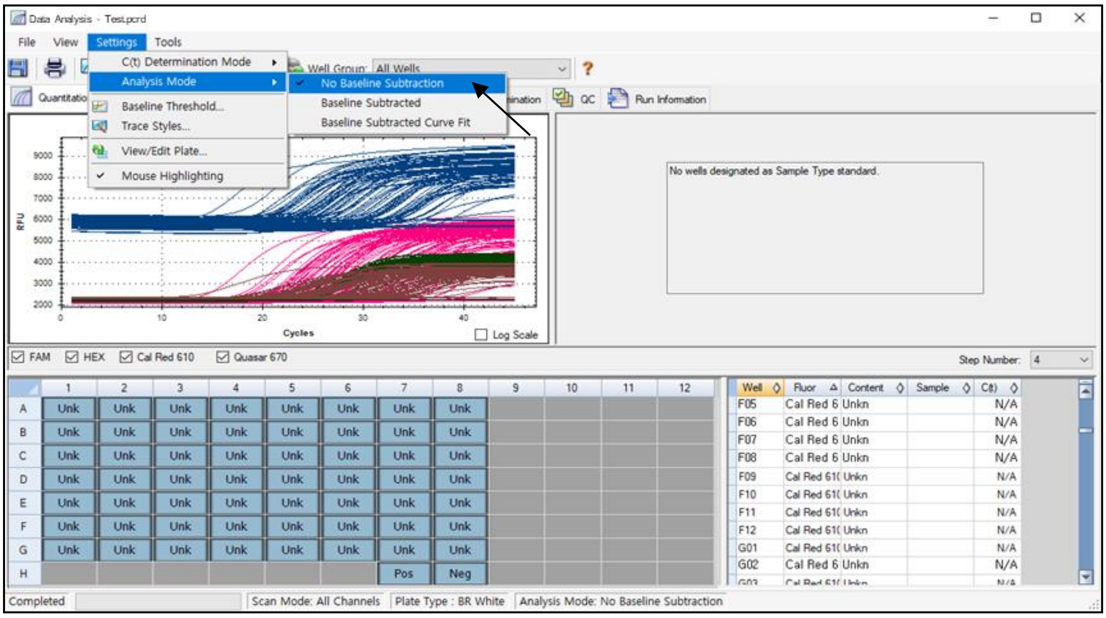

Fig. 10. **No Baseline Subtraction**

3) Select **“Seegene Export”** from Tools menu.

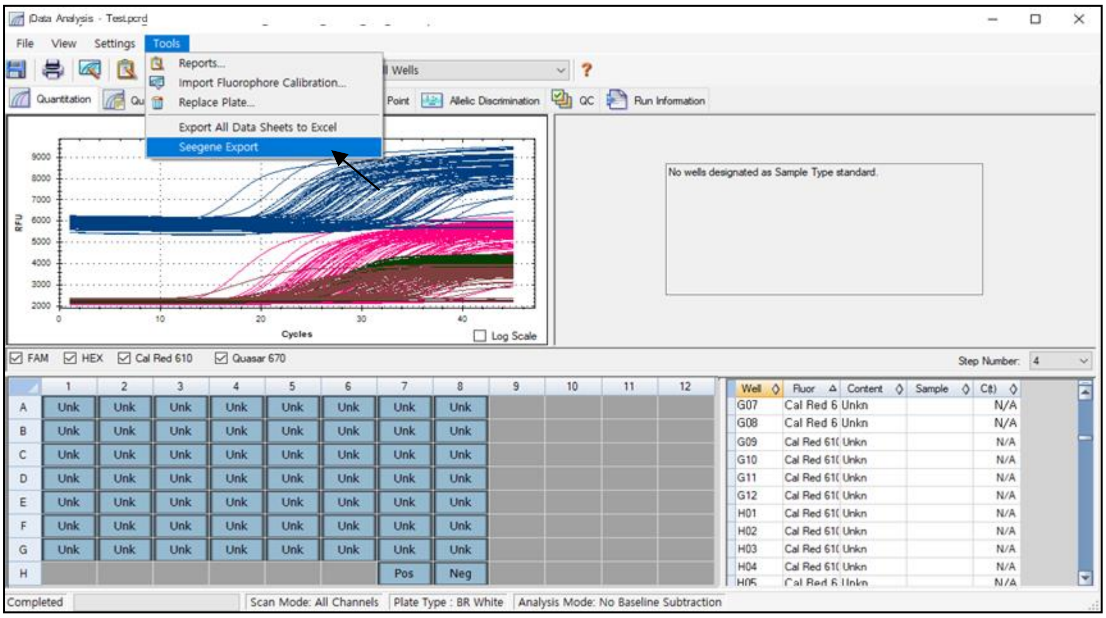

Fig. 11. **Seegene Export**

4) Choose a location to save data and click **“OK”**.

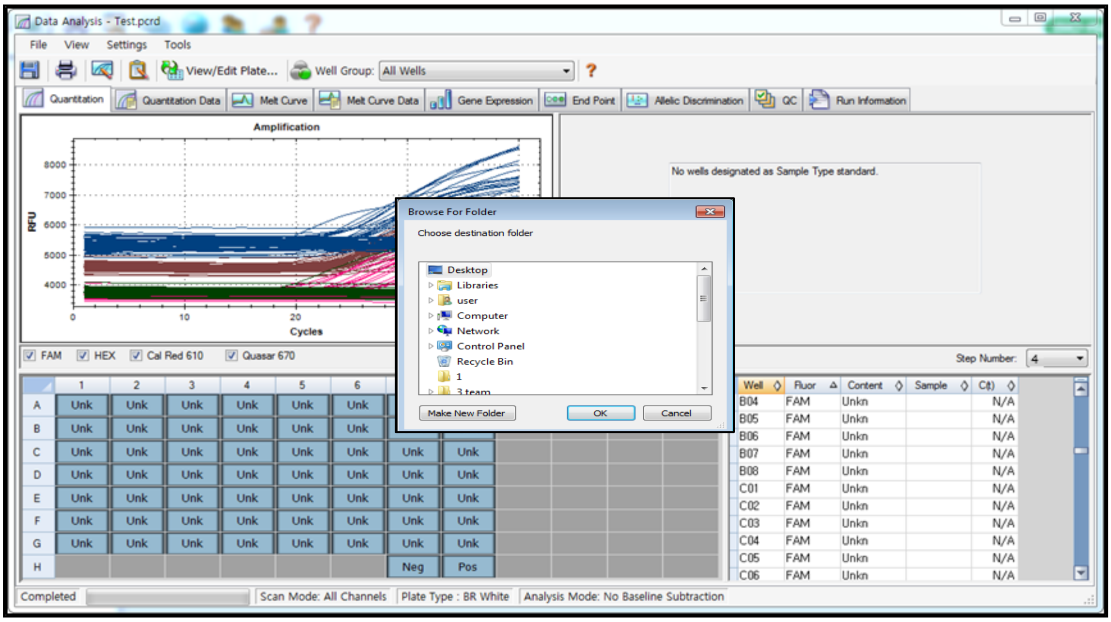

Fig. 12. **Seegene Export to designated folder**

C. Settings for Data Analysis in Seegene Viewer

1) Open Seegene Viewer program, and click **"Option"** to select ***CFX96 or CFX96 Dx*** in the **"Instrument"**.

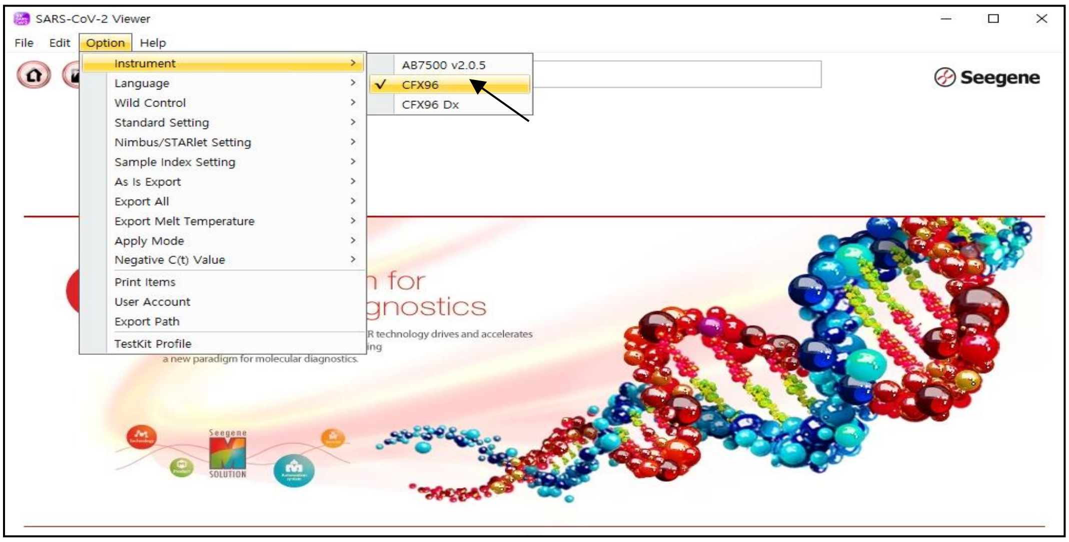

Fig. 13. **Seegene Viewer**

2) Click **"Open"** to find the saved file in folder "QuantStep4", open the results file, and select the test kit from the **"PRODUCT"** menu.

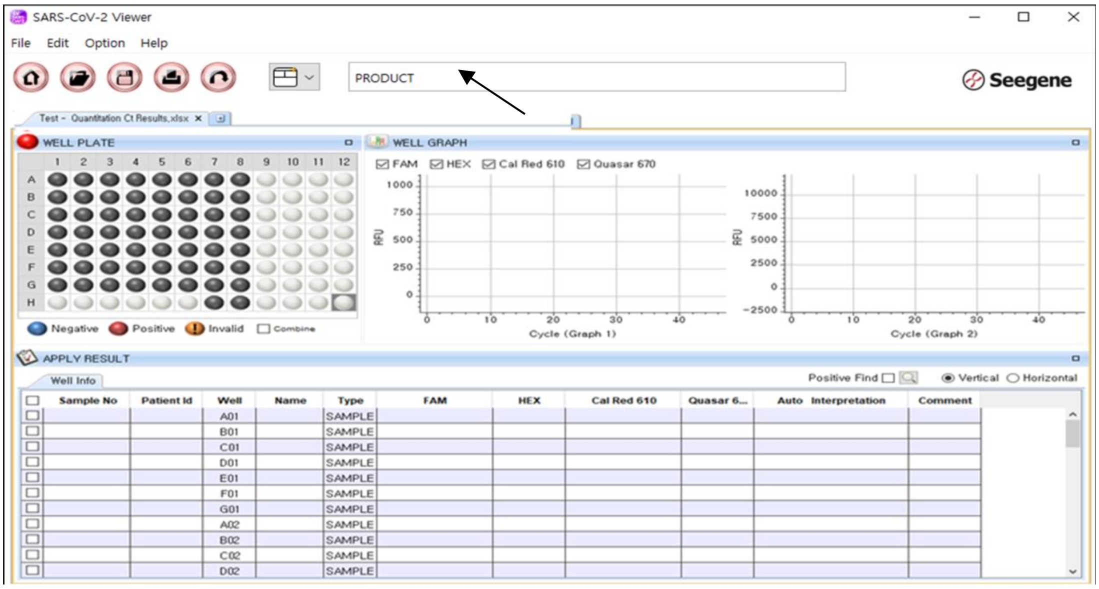

Fig. 14. **Settings for Data Analysis in Seegene Viewer**

Note: In case of extraction-free method is applied, select "Allplex™ SARS-CoV-2 Assay (extraction-free)" from the PRODUCT menu.

3) Check the result for each well.

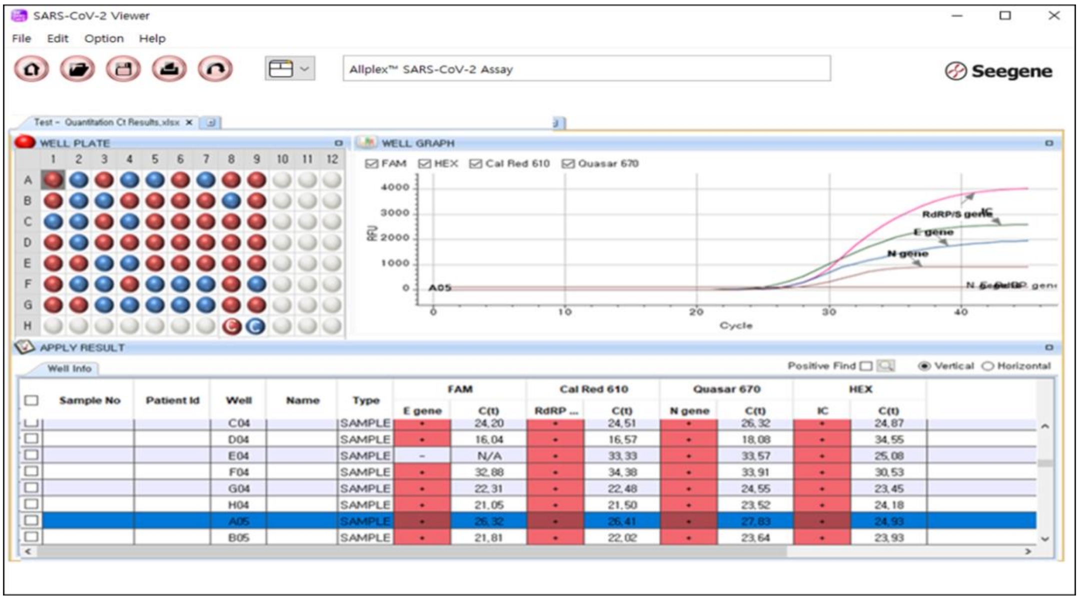

Fig. 15. **Test result on Seegene Viewer**

4) Validity Criteria of Control Results

a. Valid Assay Run

To check the validity of experiments, the PCR runs should be accompanied with PC (Positive Control) and NC (Negative Control). Assay run is determined as valid when all of the following criteria are met:

1) Standard extraction

Control	Seegene Viewer Result				
	FAM (C_t)	Cal Red 610 (C_t)	Quasar670 (C_t)	HEX (C_t)	Auto Interpretation
	E gene	RdRP/S gene	N gene	IC	
Positive Control	≤ 40	≤ 40	≤ 40	≤ 40	Positive Control(+)
Negative Control	N/A	N/A	N/A	N/A	Negative Control(-)

2) Extraction-free method

Control	Seegene Viewer Result				
	FAM (C_t)	Cal Red 610 (C_t)	Quasar670 (C_t)	HEX (C_t)	Auto Interpretation
	E gene	RdRP/S gene	N gene	IC	
Positive Control	≤ 40	≤ 40	≤ 40	≤ 40	Positive Control(+)
Negative Control	N/A	N/A	N/A	≤ 40	Negative Control(-)

b. Invalid Assay Run

In case of a validity failure, the results should not be interpreted or reported. And the PCR reaction must be repeated.

2. CFX96™ Dx System (CFX Manager™ Dx v3.1)

2.1 Real-time PCR Instrument Setup

Note: CFX96™ Dx System (Bio-Rad) experiment setup can be divided into three steps: Protocol Setup, Plate Setup, and Start Run.

A. Protocol Setup

1) In the main menu, select **"File"** → **"New"** → **"Protocol"** to open **"Protocol Editor"**.

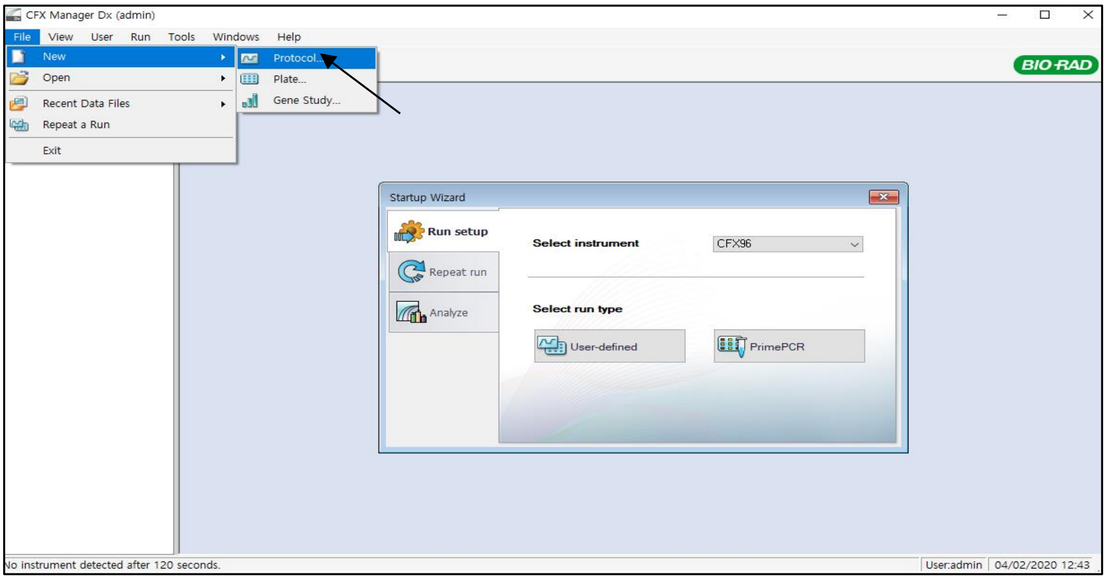

Fig. 16. **Protocol Setup**

2) In **“Protocol Editor”**, define the thermal profile as follows:

Step	No. of cycles	Temperature	Duration
1	1	50°C	20 min
2		95°C	15 min
3	45	95°C	10 sec
4*		60°C	15 sec
5*		72°C	10 sec
6	GOTO Step 3, 44 more times		

Note*: Plate Read Step. Fluorescence is detected at 60°C and 72°C.

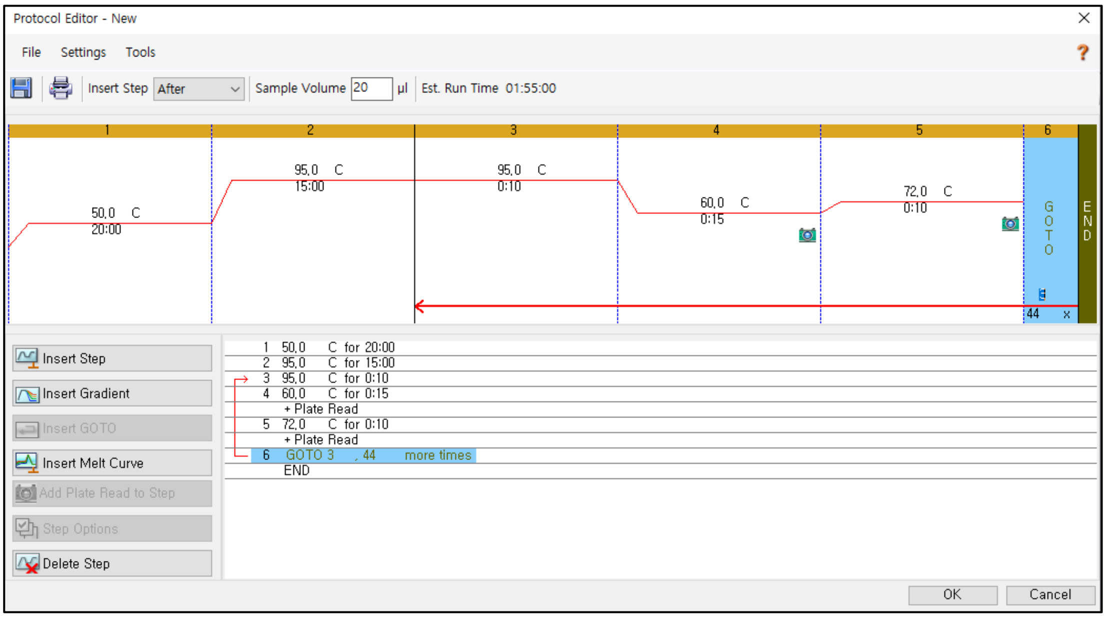

Fig. 17. **Protocol Editor**

3) Click the box next to **“Sample Volume”** to directly input 20 µL.

4) Click **“OK”** and save the protocol to open the **“Run Setup”** window.

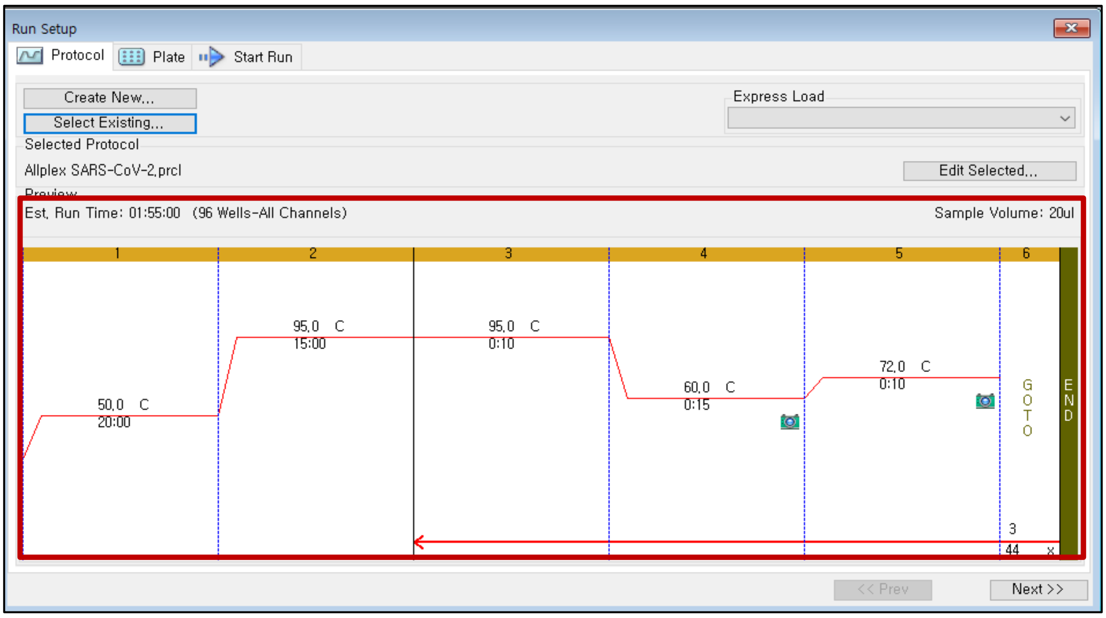

Fig. 18. **Run Setup: Protocol**

B. Plate Setup

1) From **“Plate”** tab in **“Run Setup”**, click **“Create New”** to open **“Plate Editor”** window.

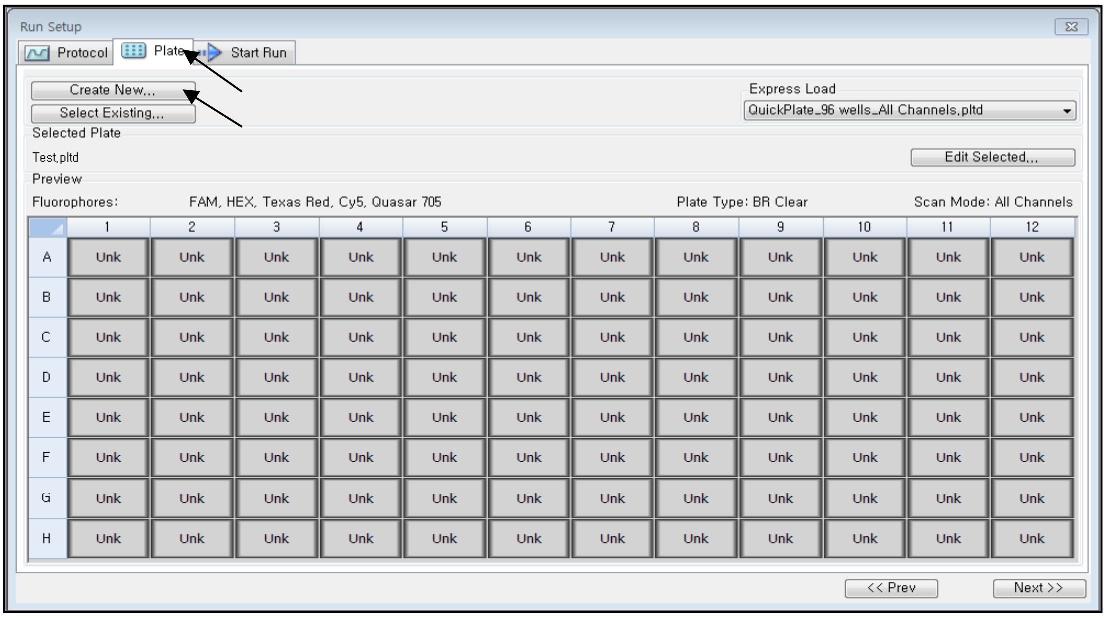

Fig. 19. **Plate Editor**

2) Click **"Select Fluorophores"** to indicate the fluorophores (***FAM***, ***HEX***, ***Cal Red 610***, ***Quasar 670***) that will be used and click **"OK"**.

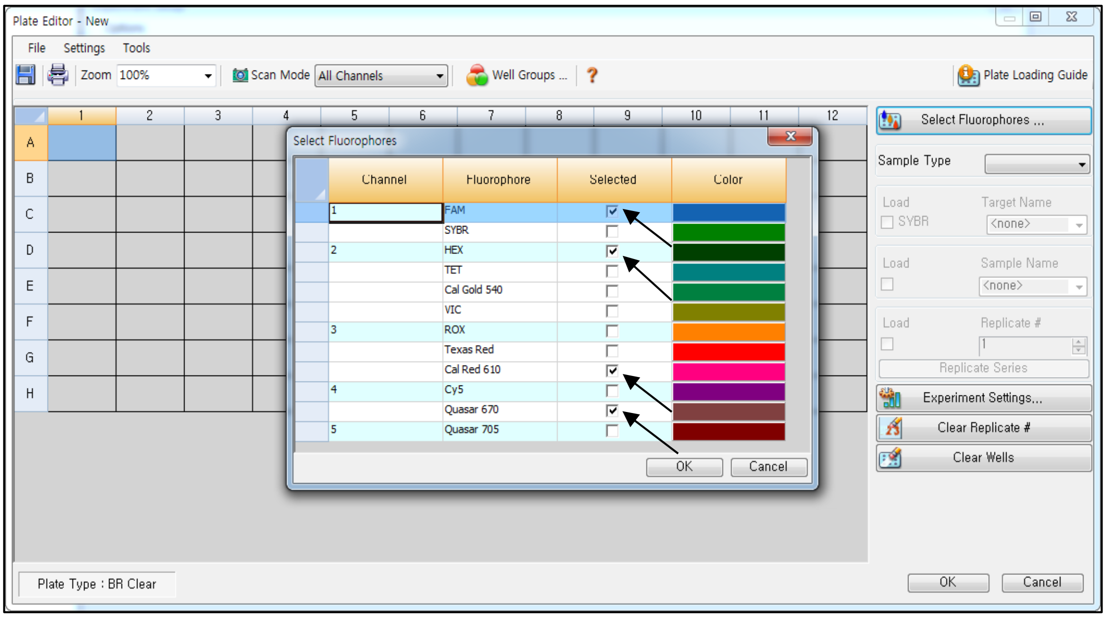

Fig. 20. **"Select Fluorophores"** (***FAM***, ***HEX***, ***Cal Red 610***, *and* ***Quasar 670***)

3) Select the wells where the PCR tube will be placed and select their sample types from the **"Sample Type"** drop-down menu.

- ***Unknown***: *Clinical samples*
- ***Negative Control***
- ***Positive Control***

4) Click on the appropriate checkboxes (***FAM, HEX, Cal Red 610, and Quasar 670***) to specify the fluorophores to be detected in the selected wells.

5) Type **"Sample Name"** and press enter key.

6) In **“Settings”** of the **“Plate Editor”** main menu, choose the **“Plate Size”** (***96 wells***) and **“Plate Type”** (***BR White***).

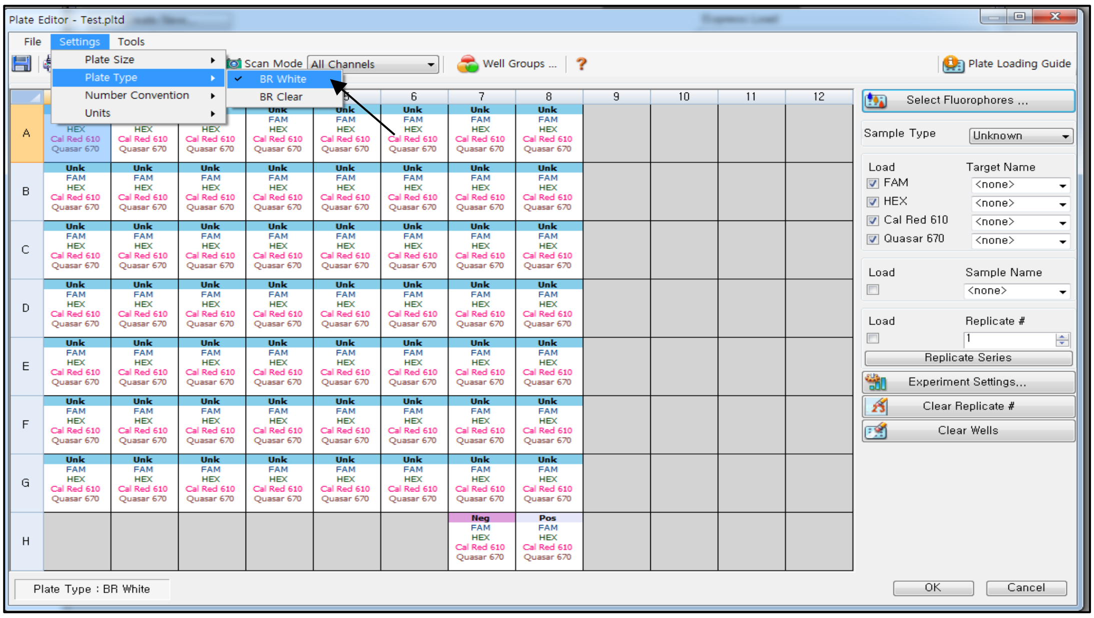

Fig. 21**. Plate Setup**

7) Click **“OK”** to save the new plate.

8) You will be returned to the **“Run Setup”** window.

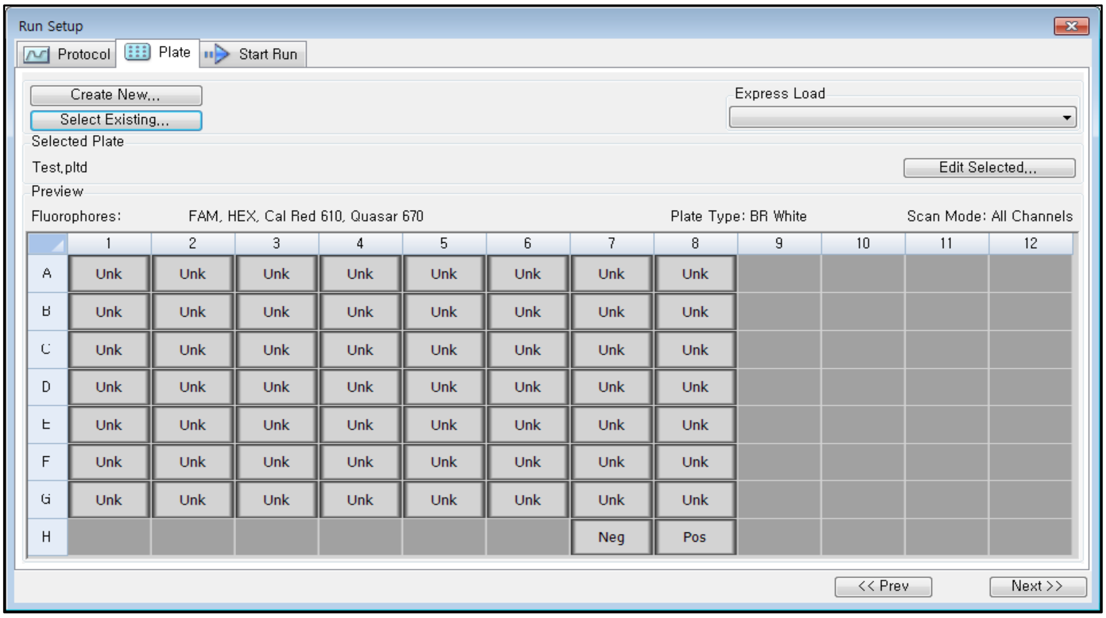

Fig. 22**. Run Setup: Plate**

9) Click **“Next”** to Start Run.

C. Start Run

1) From **“Start Run”** tab in **“Run Setup”**, click **“Close Lid”** to close the instrument lid.

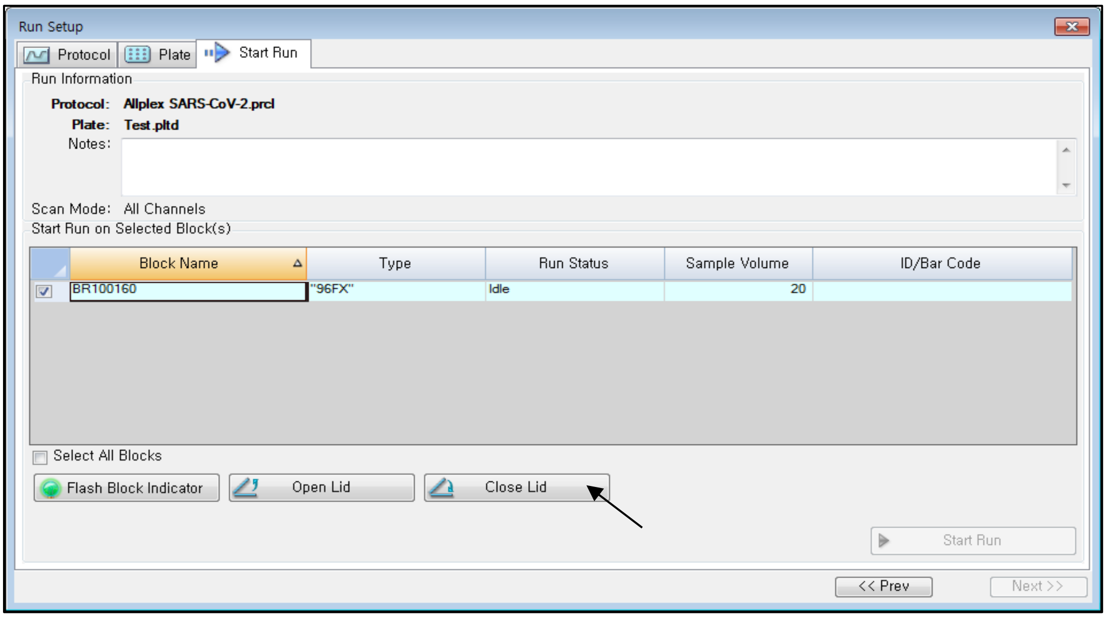

Fig. 23. **Close Lid**

2) Click **“Start Run”**.

3) Store the run file either in My Documents or in a designated folder. Input the file name, click **“SAVE”**, and the run will start.

2.2. Data Analysis

A. Create folders for data export

1) To save data of all detection steps of amplification curves from the result file, create one folder.

2) Folder name may be as desired by user (For ‘Seegene Export’ function, folders “QuantStep4” and “QuantStep5” are automatically created to save each amplification curve data under the folder created by user).

B. Pre-settings for Data Analysis in CFX96™

1) After the test, click the **“Quantification”** tab to see the amplification curve results.

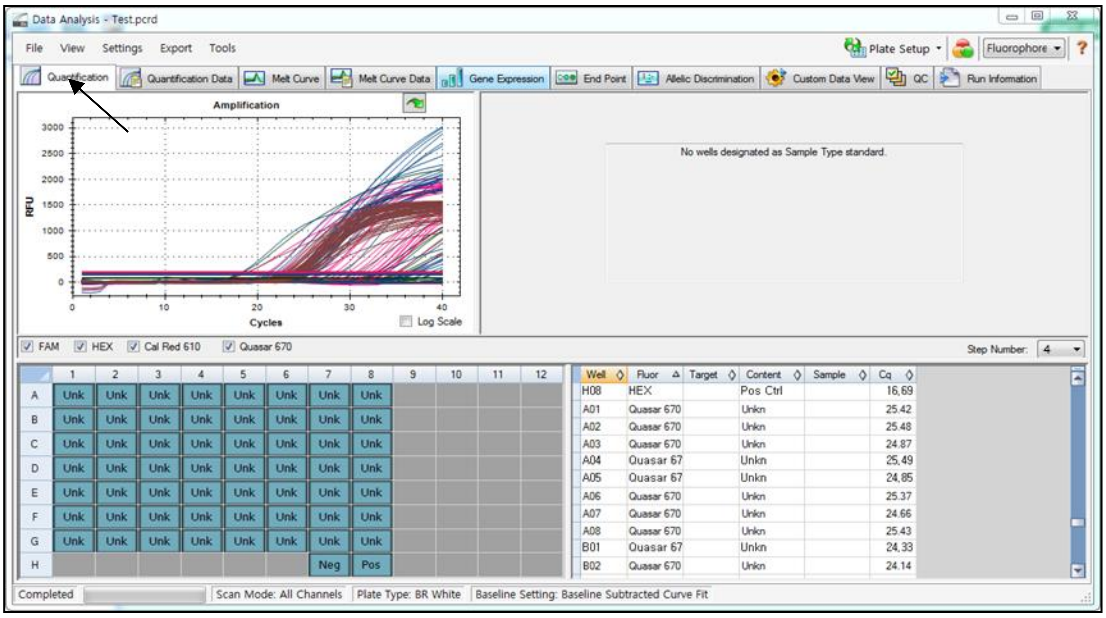

Fig. 24. **Amplification curve results**

2) Select **“No Baseline Subtraction”** from Baseline Setting of Settings menu.

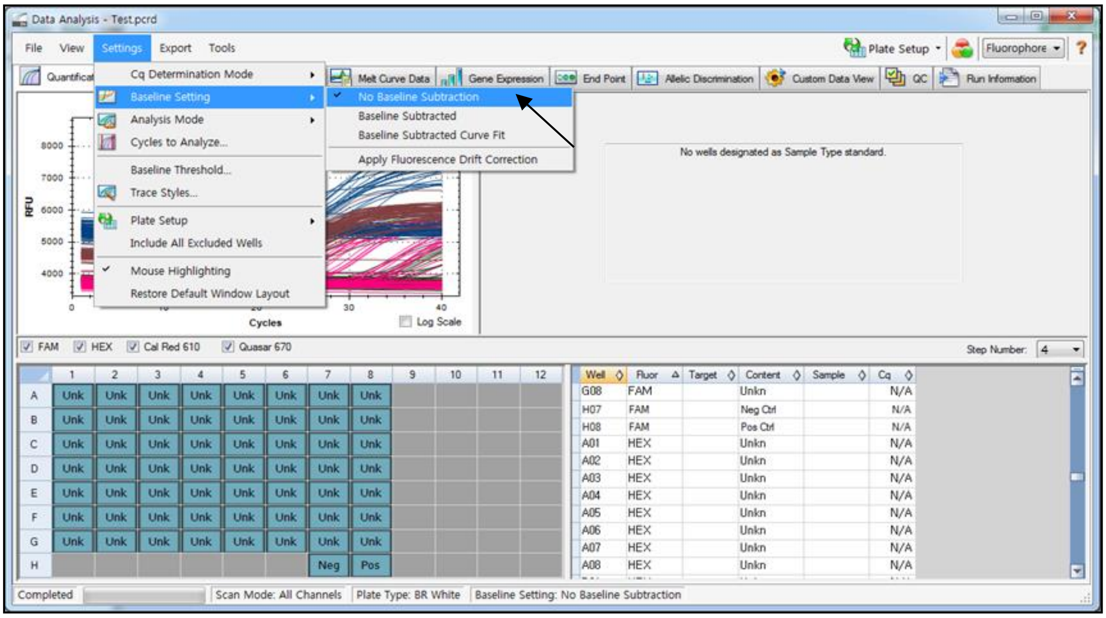

Fig. 25. **No Baseline Subtraction**

3) Select **“Seegene Export”** from Export menu.

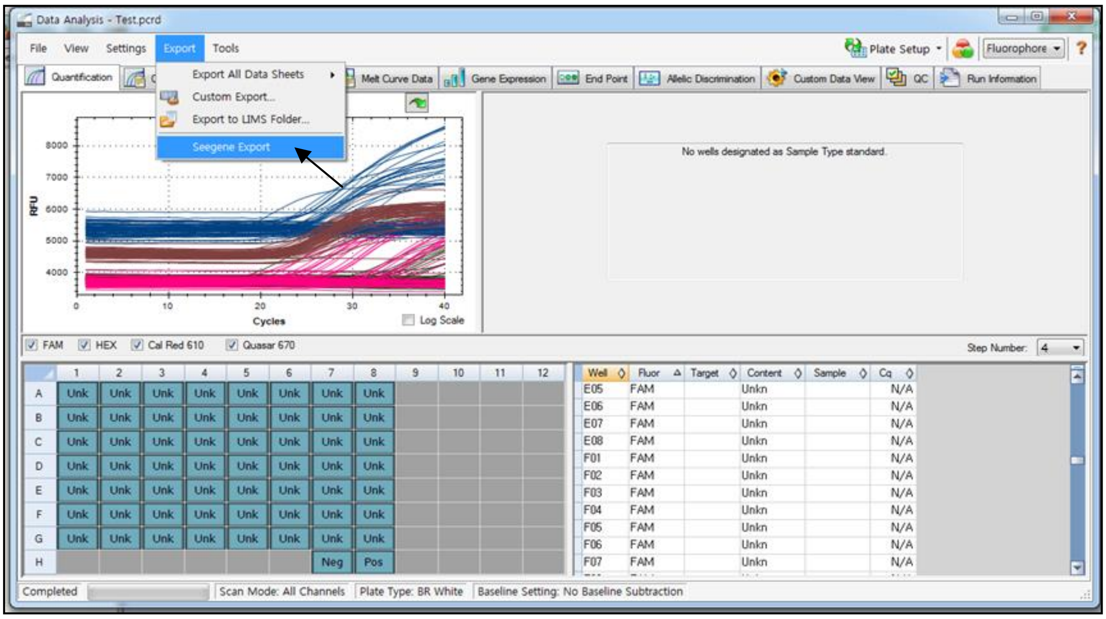

Fig. 26. **“Seegene Export”**

4) Choose a location to save data and click **“OK”**.

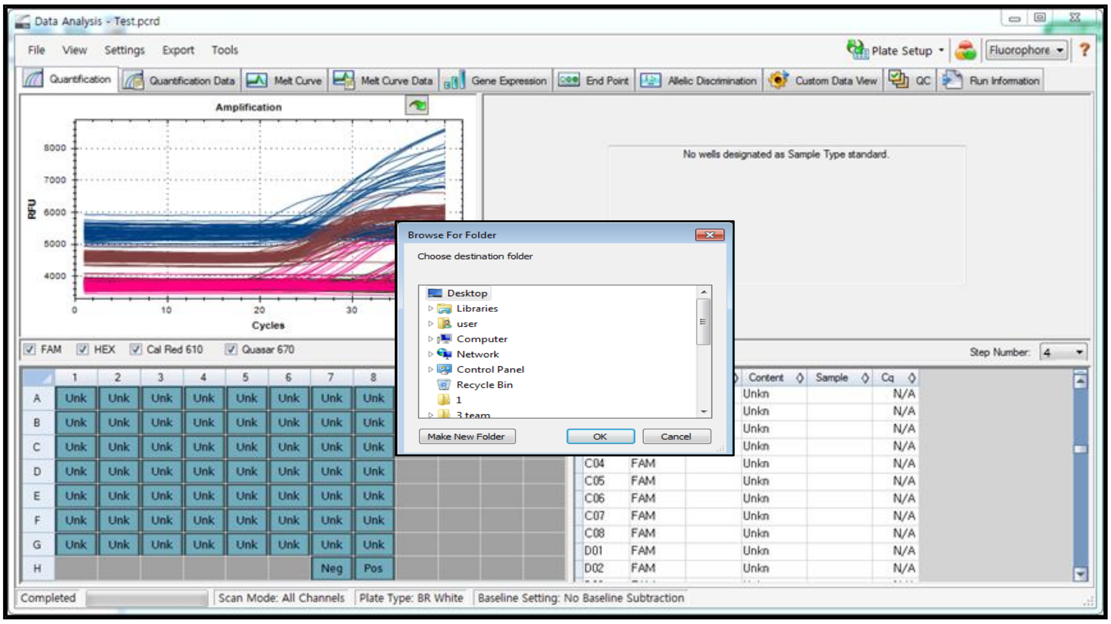

Fig. 27. **Seegene Export to designated folder**

C. Settings for Data Analysis in Seegene Viewer

1) Open Seegene Viewer program, and click **"Option"** to select ***CFX96 or CFX96 Dx*** in the **"Instrument"**.

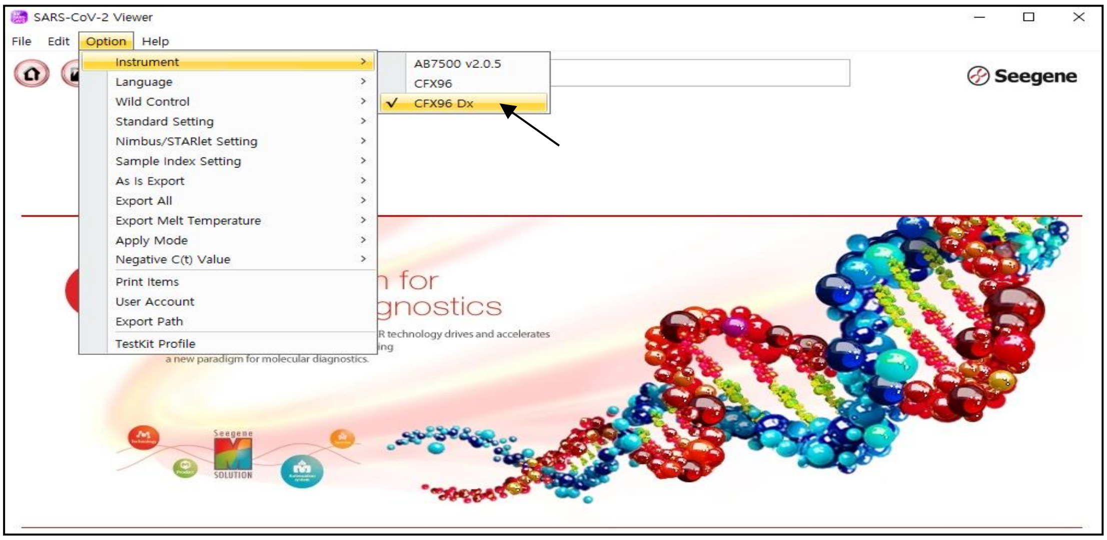

Fig. 28. **Seegene Viewer**

2) Click **"Open"** to find the saved file in folder "QuantStep4", open the results file, and select the test kit from the **"PRODUCT"** menu.

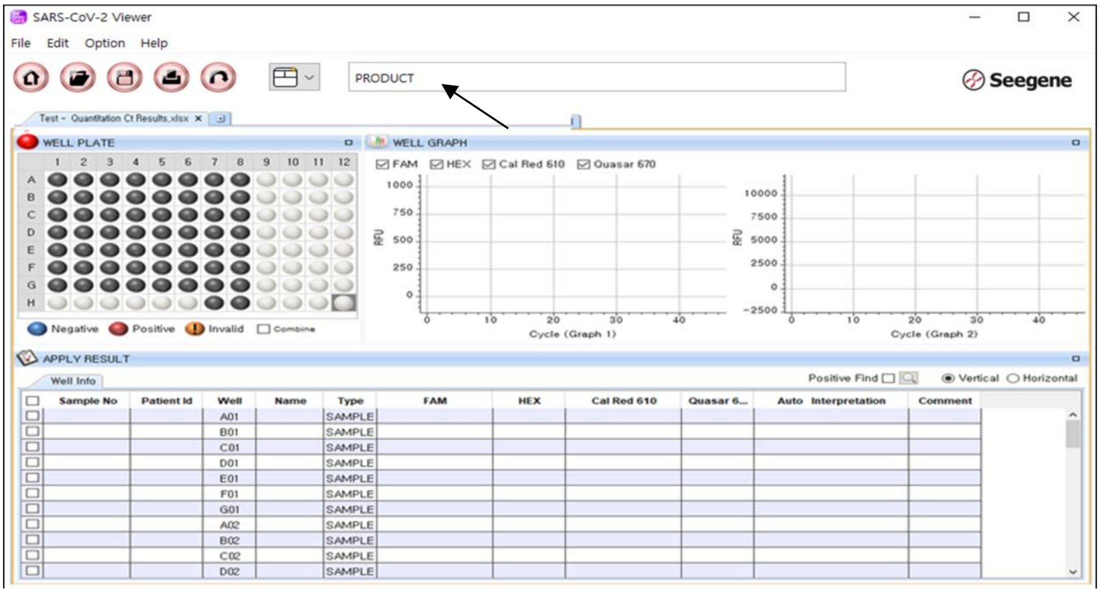

Fig. 29. **Settings for Data Analysis in Seegene Viewer**

Note: In case of extraction-free method is applied, select "Allplex^{TM} SARS-CoV-2 Assay (extraction-free)" from the PRODUCT menu.

3) Check the result for each well.

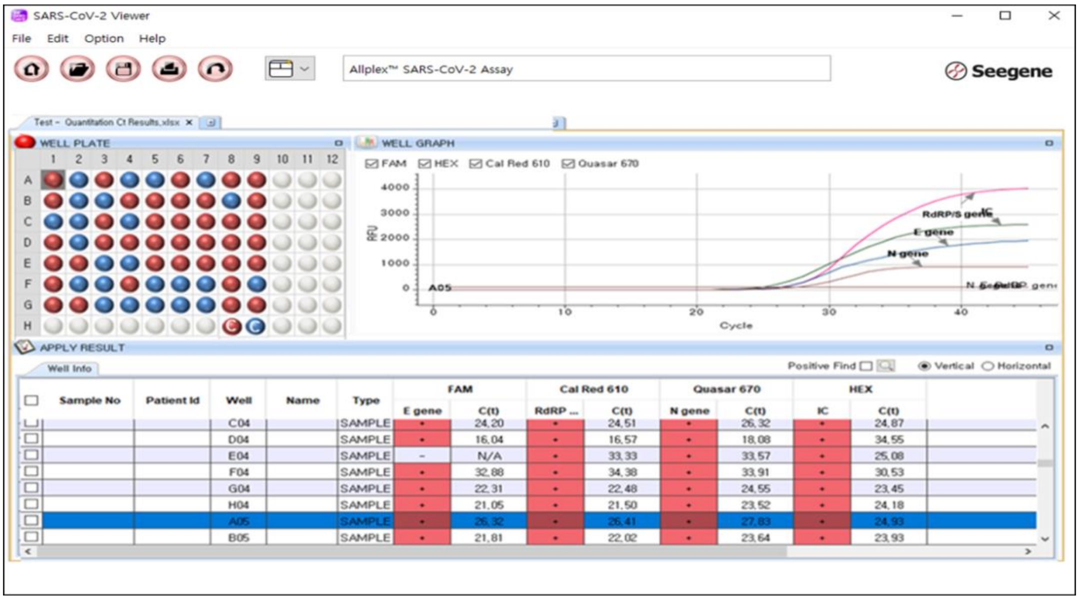

Fig. 30. **Test result on Seegene Viewer**

4) Validity Criteria of Control Results

a. Valid Assay Run

To check the validity of experiments, the PCR runs should be accompanied with PC (Positive Control) and NC (Negative Control). Assay run is determined as valid when all of the following criteria are met:

1) Standard extraction

Control	Seegene Viewer Result				
	FAM (C_t)	Cal Red 610 (C_t)	Quasar670 (C_t)	HEX (C_t)	Auto Interpretation
	E gene	RdRP/S gene	N gene	IC	
Positive Control	≤ 40	≤ 40	≤ 40	≤ 40	Positive Control(+)
Negative Control	N/A	N/A	N/A	N/A	Negative Control(-)

2) Extraction-free method

Control	Seegene Viewer Result				
	FAM (C_t)	Cal Red 610 (C_t)	Quasar670 (C_t)	HEX (C_t)	Auto Interpretation
	E gene	RdRP/S gene	N gene	IC	
Positive Control	≤ 40	≤ 40	≤ 40	≤ 40	Positive Control(+)
Negative Control	N/A	N/A	N/A	≤ 40	Negative Control(-)

b. Invalid Assay Run

In case of a validity failure, the results should not be interpreted or reported. And the PCR reaction must be repeated.

3. Applied Biosystems™ 7500 (SDS software v2.0.5)

3.1. Real-time PCR Instrument set up

Note: The instrument must be calibrated before use.

Note: Applied Biosystems™ 7500(Thermo Fisher Scientific) experiment setup can be divided into two steps: Setup and Run

A. Setup

1) In the main menu, select **“Set Up”** → **“Advanced Setup”**

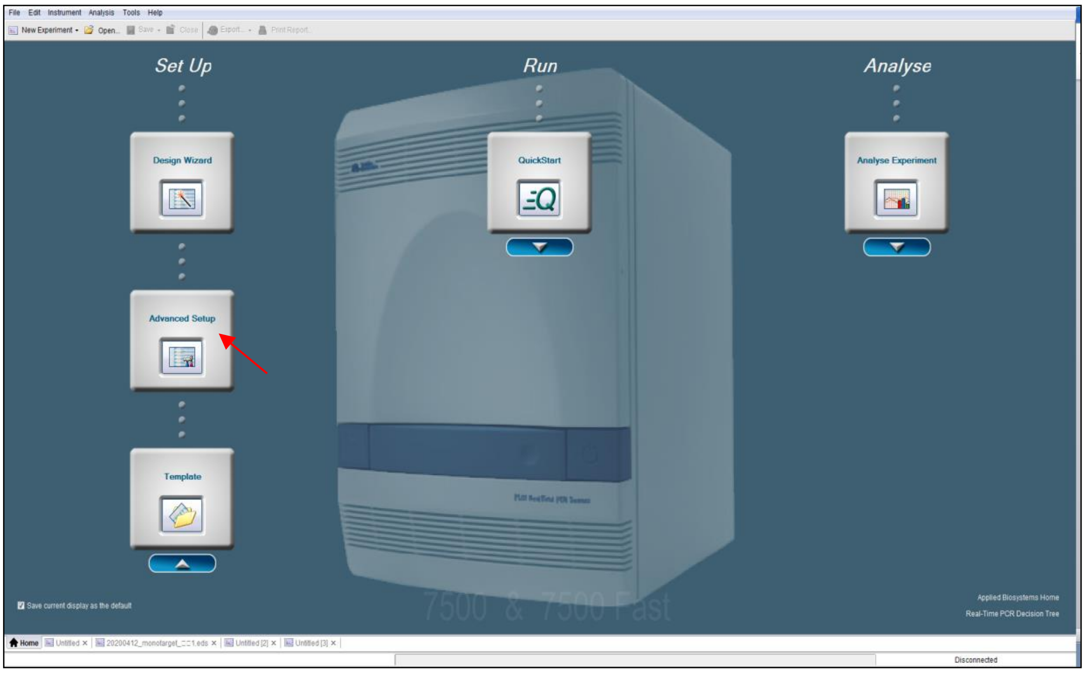

Fig 31. **Set up**

2) In the **“Experiment properties”** tab, enter **“Experiment Name”** and select Instrument, **“Experiment type”**, **“Reagents”**, and **“Ramp speed”** as follows.

**Instrument**	7500 (96 Wells)
**Experiment type**	Quantitation – Standard Curve
**Reagents**	Taqman® Reagents
**Ramp speed**	Standard

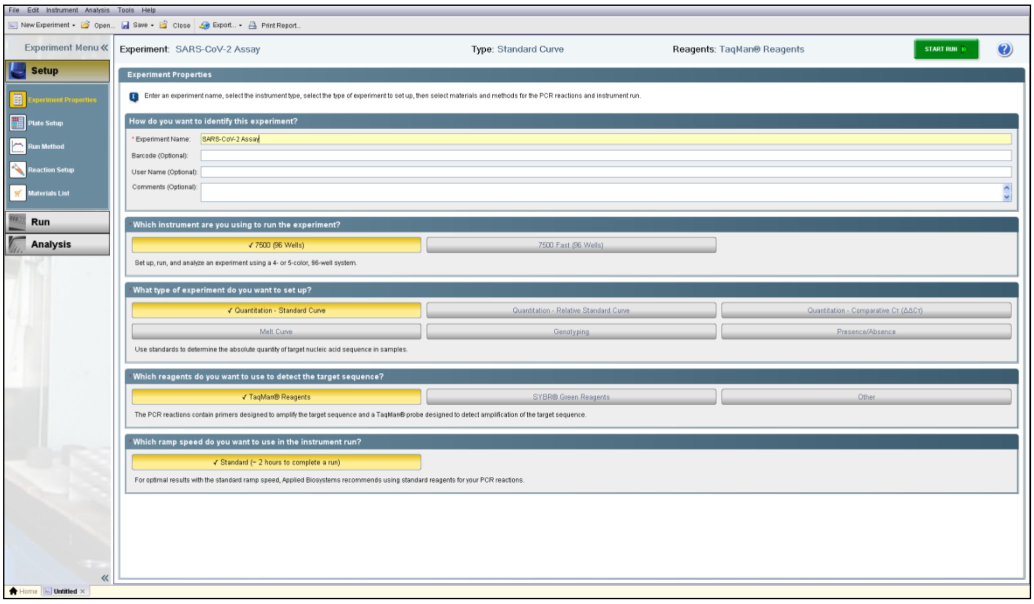

Fig. 32. **Experiment properties tab**

3) Click on **“Plate setup”** tab. In the **“Define Targets and Samples”** tab, enter “**Target Name”** and select “**Reporter”** and “**Quencher”** as follows.

Target Name	Reporter	Quencher
E gene	FAM	None
IC	VIC	None
RdRP/S gene	ROX	None
N gene	CY5	None

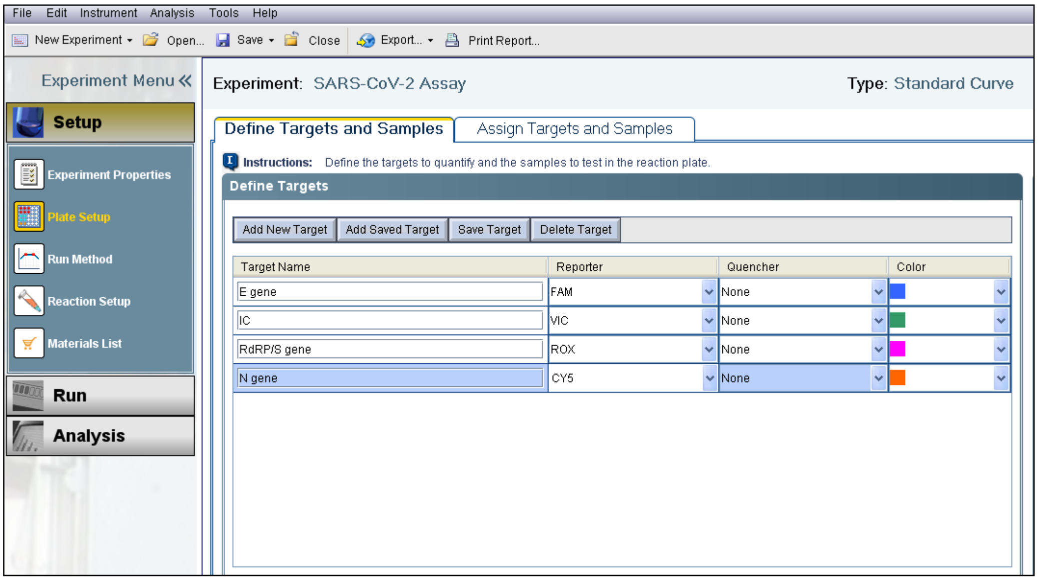

Fig. 33. **Define Targets and Samples tab**

4) Click on “**Assign Targets and Samples”** tab, select wells where the PCR tube will be placed and assign targets. Select None for Passive reference.

NOTE: If a well without sample or mastermix is selected, signal noise may be observed. Ensure that only wells containing samples or mastermix are selected.

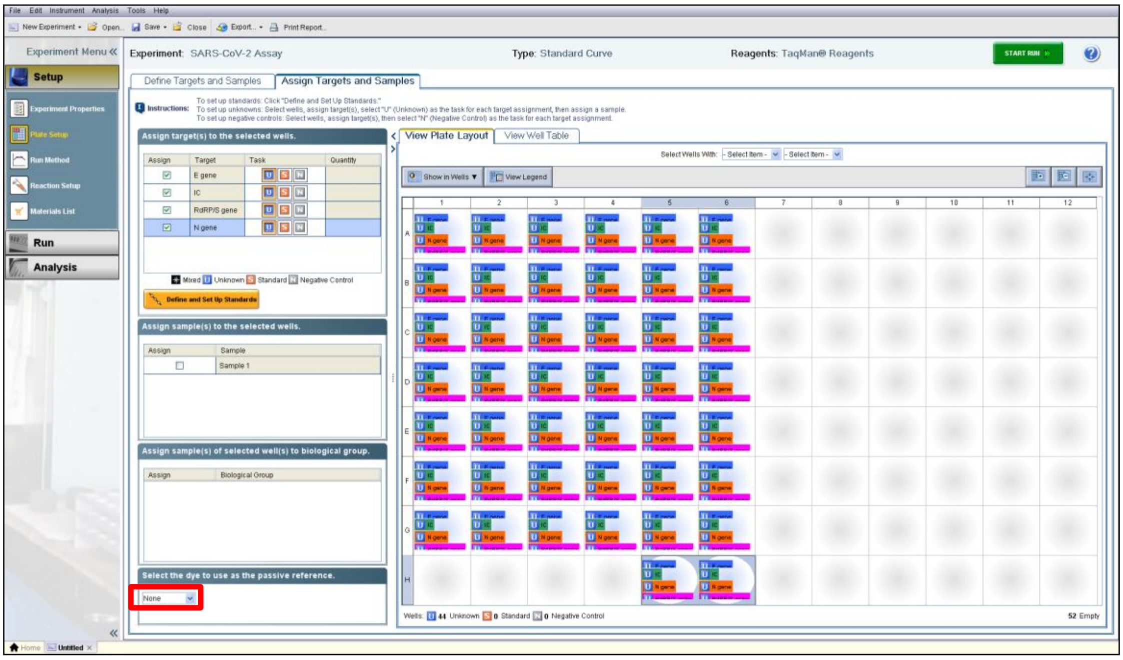

Fig. 34. **Assign Targets and Samples tab**

5) Click on “**Run Method”**. In the “**Graphical View”** or “**Tabular View tab”**, enter 20 μL as the “**Reaction Volume per Well”** field. Define the thermal profile as table below.

Step	No. of cycles	Temperature	Duration
1	1	50°C	20 min
2		95°C	15 min
3	45	95°C	10 sec
4*		60°C	30 sec
5		72°C	10 sec
6	GOTO Step 3, 44 more times		

Note*: Plate Read at **Step 4**. Fluorescence is detected at 60°C.

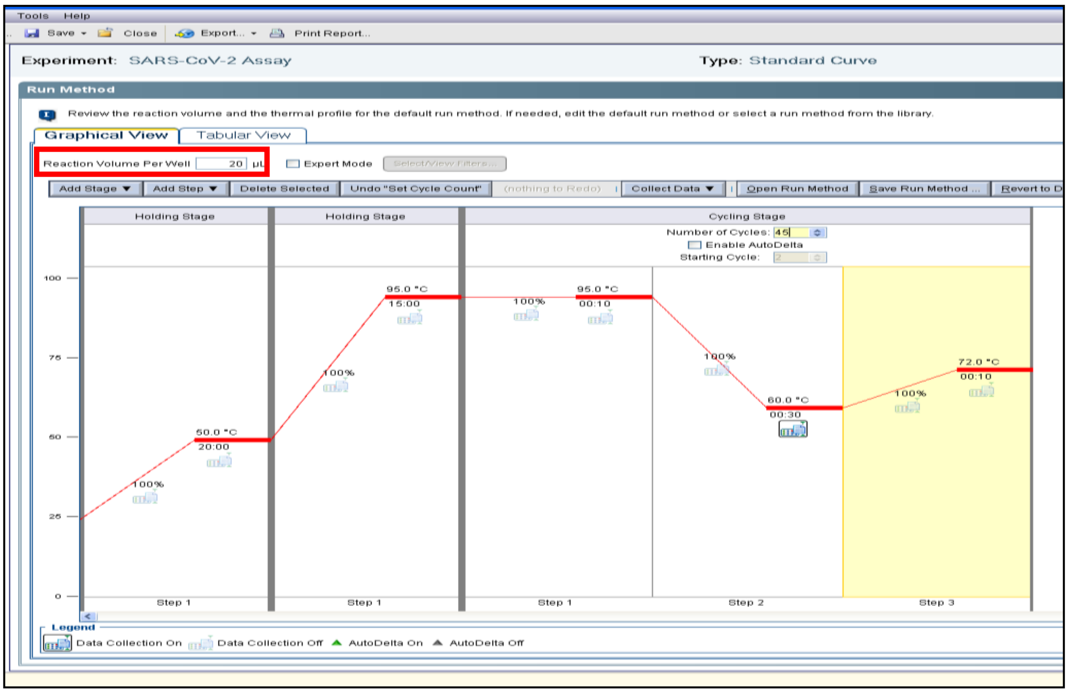

Fig. 35. **Graphical View tab**

6) Click on **"File"** → **"Save as Template"** to save the new **"template"** file in **".edt"** format. Enter the file name, select a location for the template, then click **"Save"**. The saved **"template"** can be used for future testing.

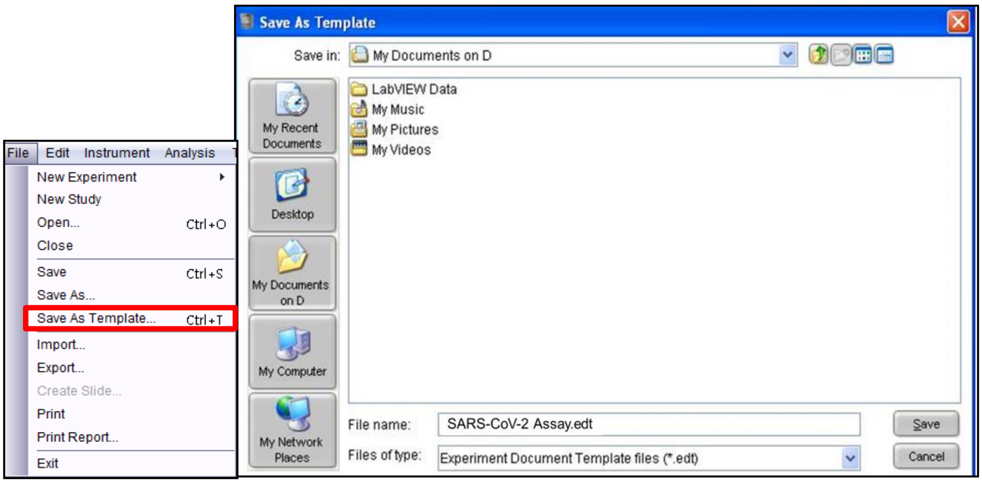

Fig. 36. **Save as Template (.edt)**

B. Run

1) Turn on the laptop and Applied Biosystems™ 7500 real-time PCR system. Ensure that the laptop is connected to the instrument.
2) Push the tray door to open the instrument. Load the PCR plate onto the plate holder of the instrument.
3) Push the tray door to close the instrument.
4) Click on **"File"** → **"Save as Template"** to save the new template file in **".eds"** format. Enter the file name, select a location for the template, then click **"Save"**.

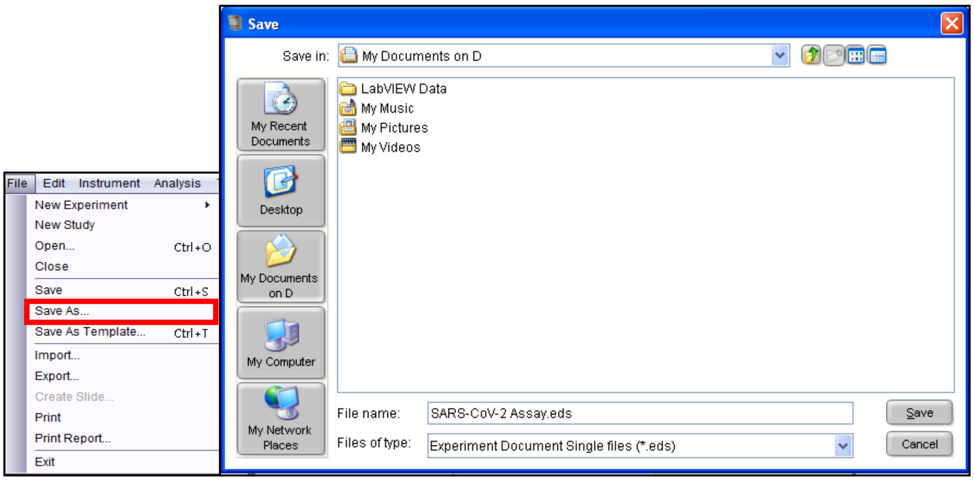

Fig. 37. **Save as (.eds)**

5) Click **START RUN.**

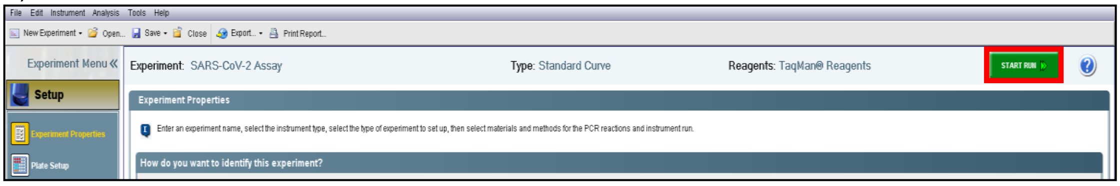

Fig. 38. **START RUN**

3.2. Data export and analysis

A. Pre-settings for Data export and analysis

1) Create a folder to save data for all of amplification curve detection steps from the result file.
2) Enter folder name as necessary.

3) Click on **“File”** → **“Export”**

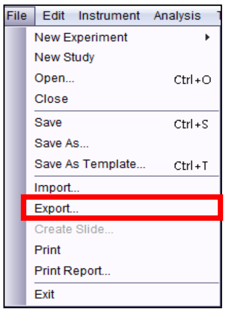

Fig. 39. **File export**

4) Click on the “**Export Properties”** tab (default) and select “**Sample Setup”, “Raw data”, “Amplification Data”, “Results”**, and “**Multicomponent Data”** under “**1. Select data to export”.**

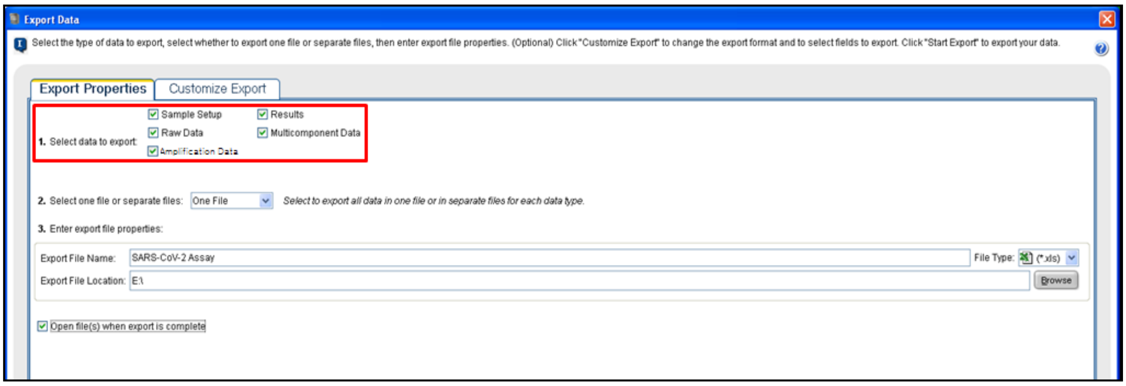

Fig. 40. **1. Select data to export**

5) Select **“One File”** under “**2. Select one or separate files:”**.

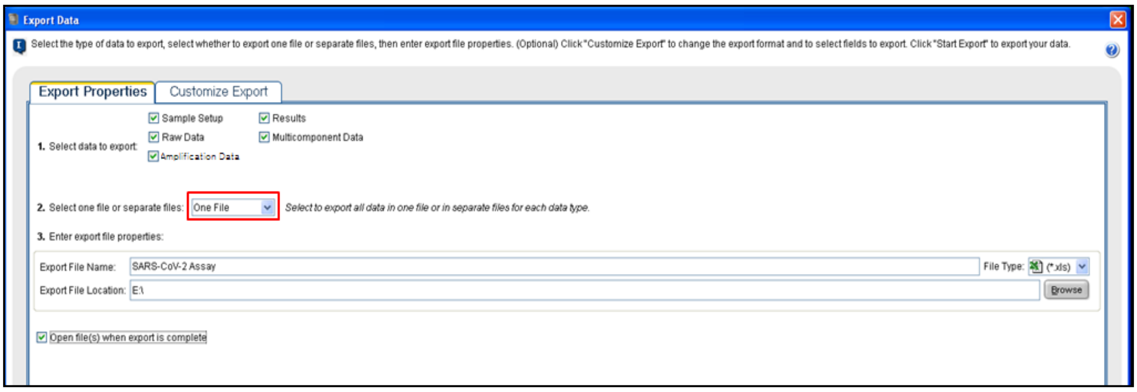

Fig. 41. **2. Select one or separate files**

6) Enter “**Export File Name”**, then select “**Export File Location”**. Select **“.xls”** in the “**File Type”** drop-down list.

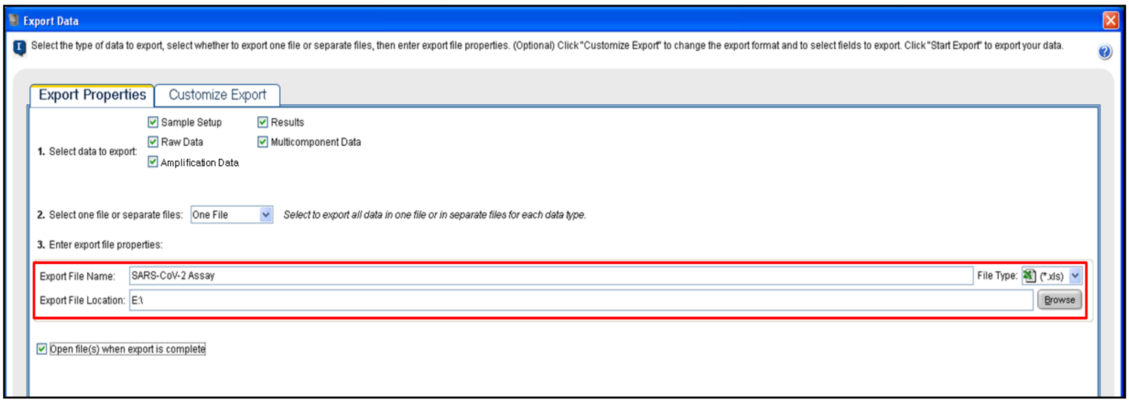

Fig. 42. **3. Enter export file properties**

7) Click **“Start Export”.**

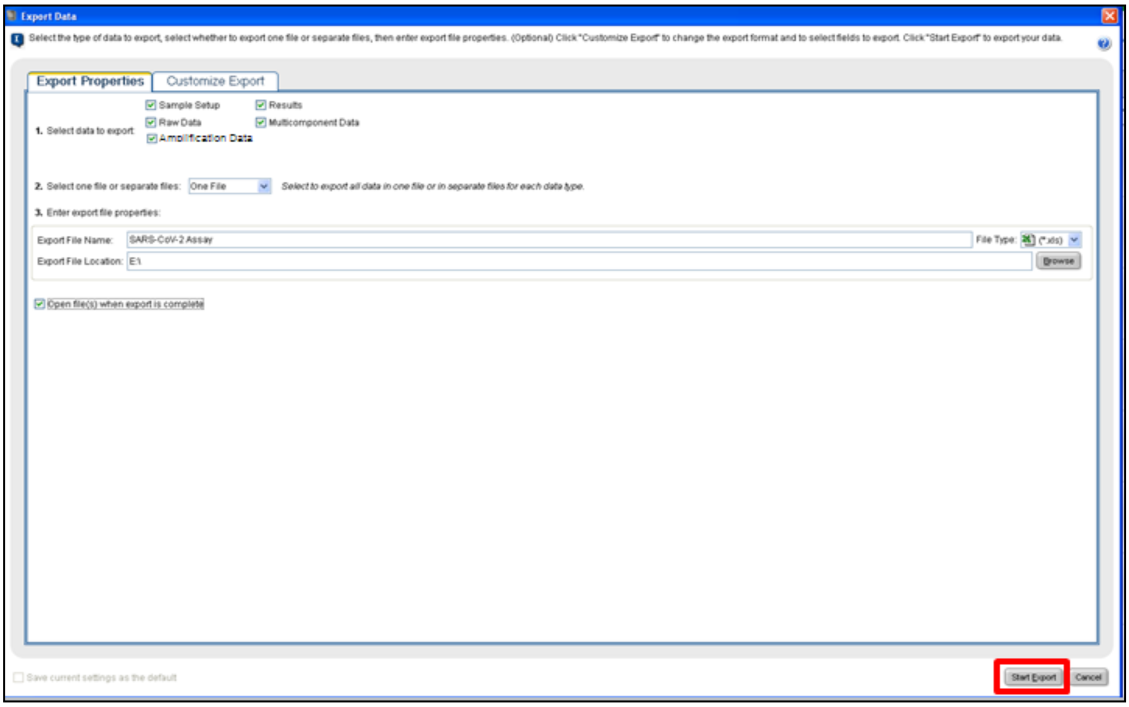

Fig. 43. **Start Export**

B. Set up for Data analysis in Seegene Viewer

1) Open the Seegene Viewer software installed on the laptop connected to the Applied Biosystems™ 7500. Click on **“Option”** to select AB7500 v2.0.5 from the **“Instrument menu”**.

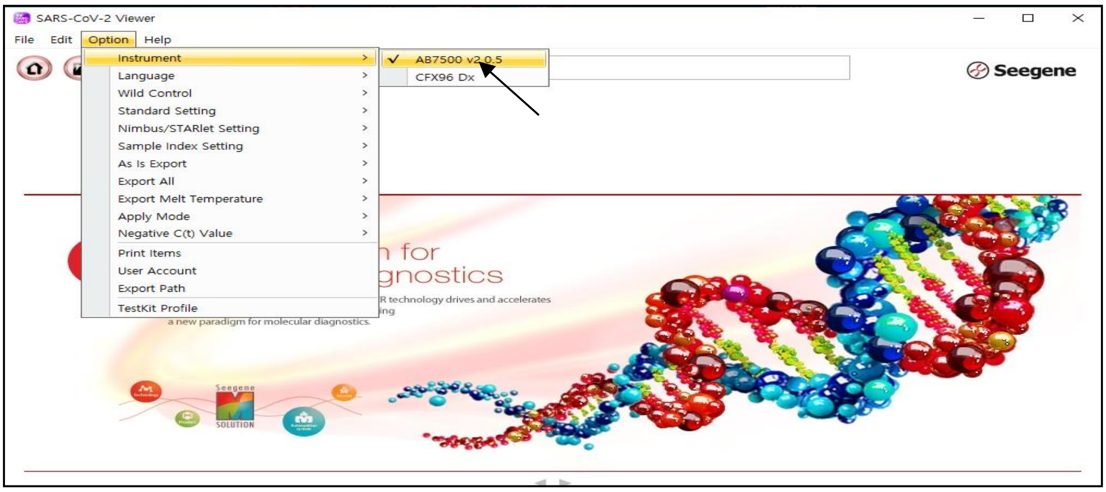

Fig. 44. **Seegene Viewer**

2) Click on the **“Open”** icon and locate the Applied Biosystems™ 7500 export data where the Applied Biosystems™ 7500 data was saved. After opening the results file, select ‘Allplex™ SARS-CoV-2 Assay’ from the PRODUCT menu.

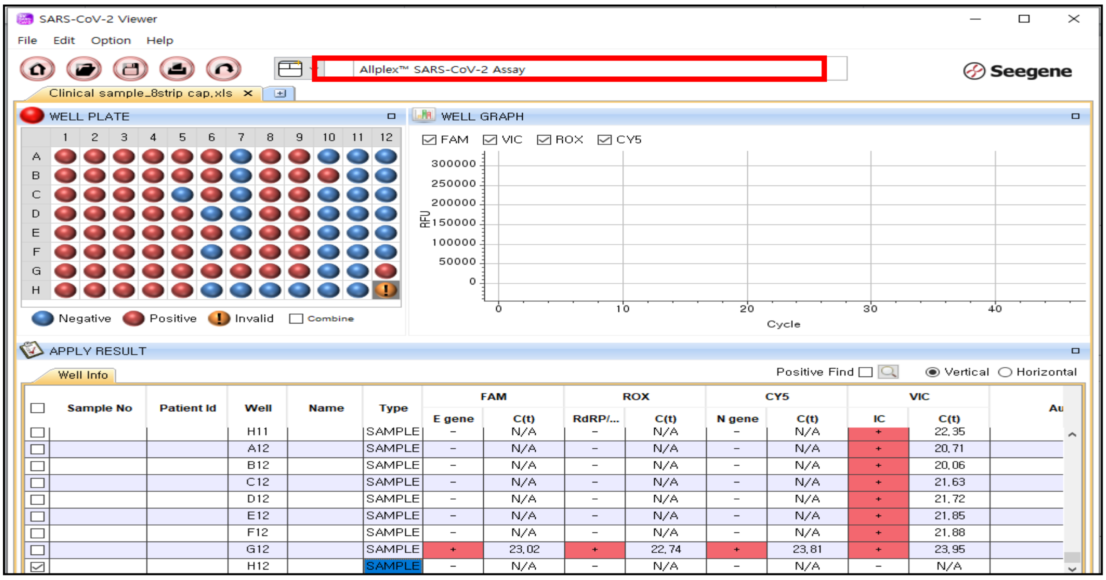

Fig. 45. **Settings for Data Analysis in Seegene Viewer**

Note: In case of extraction-free method is applied, select “Allplex^{TM} SARS-CoV-2 Assay (extraction-free)” from the PRODUCT menu.

3) Assign Positive and Negative control accordingly by selecting PC, NC under the Type drop-down menu.

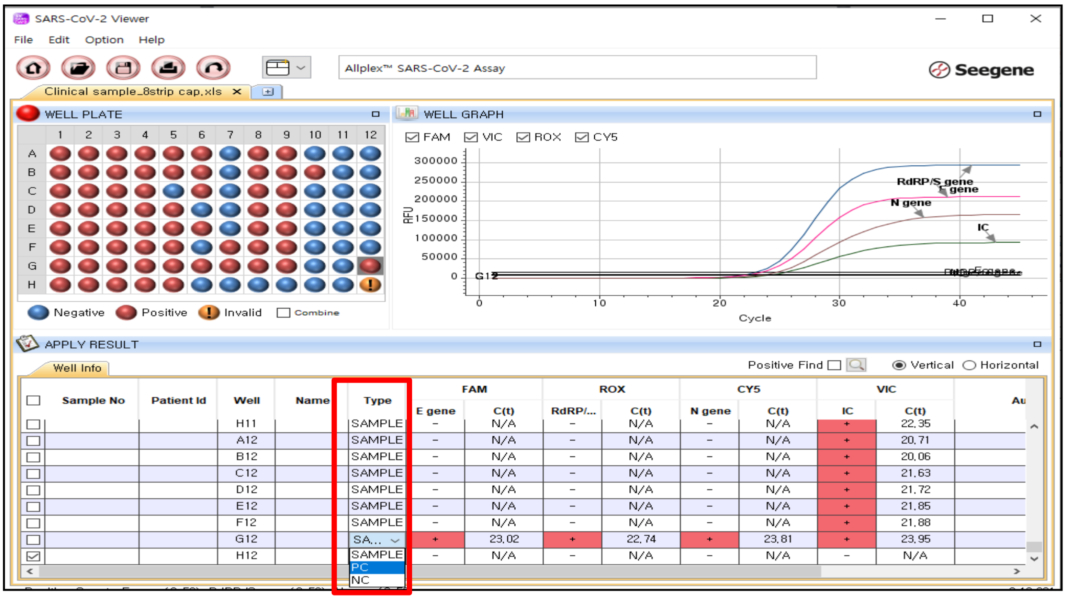

Fig. 46. **Settings for Sample Type in Seegene Viewer**

4) View test results. The auto-interpreted results for each sample can be viewed by clicking on each well.

5) Validity Criteria of Control Results

a. Valid Assay Run

To check the validity of experiments, the PCR runs should be accompanied with PC (Positive Control) and NC (Negative Control). Assay run is determined as valid when all of the following criteria are met:

1) Standard extraction

Control	Seegene Viewer Result				
	FAM (C_t)	ROX (C_t)	CY5 (C_t)	VIC (C_t)	Auto Interpretation
	E gene	RdRP/S gene	N gene	IC	
Positive Control	≤ 40	≤ 40	≤ 40	≤ 40	Positive Control(+)
Negative Control	N/A	N/A	N/A	N/A	Negative Control(-)

2) Extraction-free method

Control	Seegene Viewer Result				
	FAM (C_t)	ROX (C_t)	CY5 (C_t)	VIC (C_t)	Auto Interpretation
	E gene	RdRP/S gene	N gene	IC	
Positive Control	≤ 40	≤ 40	≤ 40	≤ 40	Positive Control(+)
Negative Control	N/A	N/A	N/A	≤ 40	Negative Control(-)

b. Invalid Assay Run

In case of a validity failure, the results should not be interpreted or reported. And the PCR reaction must be repeated.

RESULTS

1. Analyte Information

CFX96	AB7500	Analytes
Fluorophores	Fluorophores	
FAM	FAM	E gene
HEX	VIC	IC
Cal Red 610	ROX	RdRP gene, S gene
Quasar 670	CY5	N gene

2. Interpretation of Results

Analytes	C_t value	Result
Targets	≤ 40	Detected (+)
	> 40 or N/A	Not detected (-)
IC	≤ 40	Detected (+)
	> 40 or N/A	Not detected (-)

<table>
<tr><th colspan="3">Target Result</th><th rowspan="2">IC Result*</th><th rowspan="2">Auto-interpretation</th><th rowspan="2">Description</th></tr>
<tr><th>E gene</th><th>RdRP/S gene</th><th>N gene</th></tr>
<tr><td>+</td><td>+</td><td>+</td><td>+/-</td><td rowspan="6">SARS-CoV-2</td><td>All Target Results were valid. Result for SARS-CoV-2 RNA is detected.</td></tr>
<tr><td>+</td><td>-</td><td>+</td><td>+/-</td><td rowspan="5">- All Target Results were valid. Result for SARS-CoV-2 RNA is detected.
- Negative target results are suggestive of
1) A sample at concentrations near or below the limit of detection of the test,
2) A mutation in the corresponding target region,
3) Other factors.</td></tr>
<tr><td>-</td><td>+</td><td>+</td><td>+/-</td></tr>
<tr><td>+</td><td>+</td><td>-</td><td>+/-</td></tr>
<tr><td>-</td><td>+</td><td>-</td><td>+/-</td></tr>
<tr><td>-</td><td>-</td><td>+</td><td>+/-</td></tr>
<tr><td>+</td><td>-</td><td>-</td><td>+/-</td><td>SARS-CoV-2 Presumptive positive</td><td>- All Target Results were valid. Result for Sarbecovirus RNA is detected. Result for SARS-CoV-2 RNA is Presumptive Positive.
- Negative target results are suggestive of
1) A sample at concentrations near or below the limit of detection of the test,
2) A mutation in the corresponding target region,
3) Other factors.
- Repeat test with more nucleic acid (up to 10ul)
- For samples with the same result on the repeated test, additional confirmatory testing may be conducted, if it is necessary to</td></tr>
</table>

					differentiate between SARS-CoV-2 and other Sarbecovirus currently unknown to infect humans, for epidemiological purposes or clinical management.
-	-	-	+	**Not detected (-)**	All target results were valid. Result for SARS-CoV-2 RNA is not detected.
-	-	-	-	**Invalid****	- Results suggest inadequate specimen collection or processes (*e.g.,* no exogenous IC added) or the presence of PCR inhibitors.<br>- Repeat the test from the nucleic acid extraction using another aliquot of the original specimen.<br>- If the same result is shown in the diluted nucleic acid, please collect samples again.

* High level of target nucleic acids may cause interference in Internal Control detection and readout. Invalid IC signal does not indicate that the positive results for targets are invalid.

** See TROUBLESHOOTINGS section for the detailed instruction.

3. Application to Clinical Samples

Clinical Sample 1

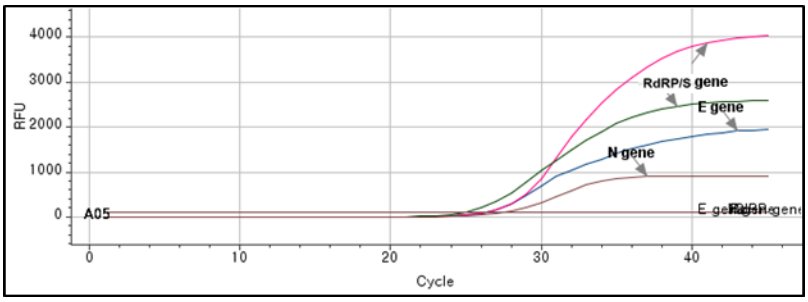

Clinical Sample 2

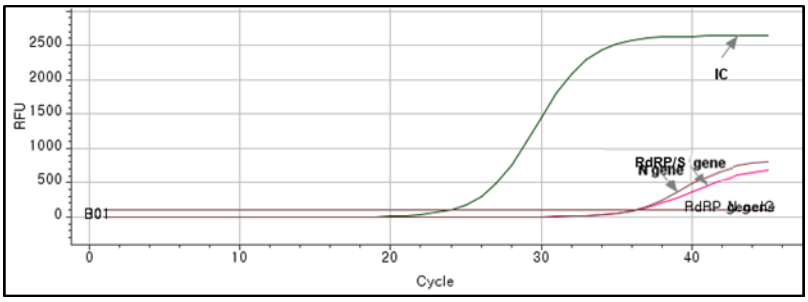

CFX96	FAM		Cal Red 610		Quasar 670		HEX		Auto Interpretation
AB7500	FAM		ROX		CY5		VIC		
Sample	E gene	C(t)	RdRP/ S gene	C(t)	N gene	C(t)	IC	C(t)	
1	+	26.32	+	26.41	+	27.83	+	24.93	SARS-CoV-2
2	-	N/A	+	36.25	+	36.36	+	23.85	SARS-CoV-2

TROUBLESHOOTING

Allplex™ SARS-CoV-2 Assay		
**OBSERVATION**	**PROBABLE CAUSES**	**SOLUTION**
**No signal**	The fluorophores for data analysis do not comply with the protocol	Select the correct fluorophores for data analysis.
	Incorrect setting of real-time thermal cycler	Please check the thermal cycling conditions and repeat the test under the correct settings.
	Incorrect storage or expiration of the test kit	Please check the storage conditions (See page 8) and the expiry date (refer to label) of the test kit and use a new kit if necessary.
	Nucleic acid extraction failure	If IC had been exogenously added to the specimen prior to extraction, the absence of IC signal may indicate loss of nucleic acids during the extraction. Make sure to use recommended extraction method.<br>If due to inhibitors, re-extract the original specimen or the specimen may be diluted with saline buffer 1/3~1/10 fold and then add "RP-V IC 2" to the diluted specimen.
**No Internal Control signal**	High load of pathogen's nucleic acid	If target pathogen signal is observed but not IC, then IC amplification may have been inhibited by high titer of target pathogen. If you want to observe IC signal, dilute the specimen (1/3~1/10) in saline buffer and repeat the test from extraction step.
	Presence of PCR Inhibitor	Please dilute the specimen (1/3~1/10) in saline buffer and repeat the test from extraction step.

Allplex™ SARS-CoV-2 Assay		
**OBSERVATION**	**PROBABLE CAUSES**	**SOLUTION**
**Putative false positive or target signal(s) observed in Negative Control**	Contamination	Decontaminate all surfaces and instruments with sodium hypochlorite and ethanol. Only use filter tips throughout the procedure and change tips between tubes. Repeat the entire procedure from nucleic acid extraction with the new set of reagents.
**Putative False negative or no signal observed in Positive Control**	Error in specimen collection	Please check the specimen collection method, and re-collect the specimen.
	Incorrect storage of the specimen	Please re-collect the specimen and repeat the entire procedure. Ensure that the specimen is stored as recommended.
	Error in nucleic acid extraction	Please check the nucleic acid extraction procedure as well as nucleic acid concentration, and re-extract the nucleic acid.
	Error in adding nucleic acid to correct PCR tubes	Check the sample numbers of tubes containing nucleic acid and make sure to add nucleic acid into the correct PCR tubes and carefully repeat the test if necessary.
	Presence of inhibitor	Please dilute the specimen (1/3~1/10) in saline buffer and repeat the test from extraction step.
	Incorrect PCR mixture	Confirm that all components are added to the reaction mixture (Sensitivity is compromised with pre-composed premix). All reagents must be homogenized and spun down before use.
**Spikes in any cycles of amplification curve**	Bubble in the PCR tube	Centrifuge the PCR tube before run.
**No signal from extraction-free method**	Presence of PCR Inhibitor	Please check the specimen collection device. ENAT PM 2ML PERNASAL APPLICATOR, GeneTM Set (GTS2) and GeneTM Set (GTS1) are not applicable to extraction-free method.

PERFORMANCE

1. Specificity

The high specificity of Allplex™ SARS-CoV-2 Assay is ensured by the oligos designed specifically for the targets of interest. Allplex™ SARS-CoV-2 Assay was tested for cross-reactivity to 65 different pathogens, and PCR amplification and detection were only identified for the specified targets.

NO.	Organism	Source	Isolate No.	Result†
1	SARS-CoV-2 isolate Australia/VIC01	TWIST BIOSCIENCE	102019	E, RdRP/S, N gene Detected
2	SARS-CoV-2 isolate Wuhan-Hu-1	TWIST BIOSCIENCE	102024	E, RdRP/S, N gene Detected
3	Human coronavirus HKU1	Korean isolate		Not Detected
4	Human coronavirus OC43	ATCC	VR-1558	Not Detected
5	Human coronavirus NL63	ZMC	0810228CF	Not Detected
6	Human coronavirus 229E	Korean isolate		Not Detected
7	SARS-coronavirus	ZMC	NATSARS-ST	E gene Detected
8	MERS-coronavirus	ZMC	NATMERS-ST	Not Detected
9	Influenza A virus (H1N1)	ATCC	VR-95 (H1N1)	Not Detected
10	Influenza A virus (H3N2)	ATCC	VR-547	Not Detected
11	Influenza B virus	ATCC	VR-523	Not Detected
12	Human Rhinovirus 1	KBPV	VR-81	Not Detected
13	Rhinovirus 21	KBPV	VR-40	Not Detected
14	Human rhinovirus type 90	ATCC	VR-1291	Not Detected
15	Human rhinovirus type 16	ATCC	VR-283	Not Detected
16	Human rhinovirus type 42	ATCC	VR-338	Not Detected
17	Human rhinovirus type 8	ATCC	VR-488	Not Detected
18	Human rhinovirus type 14	ATCC	VR-284	Not Detected
19	Human enterovirus type 68	ATCC	VR-1826	Not Detected
20	Human enterovirus type 70	ATCC	VR-836	Not Detected
21	Human enterovirus type 71	ATCC	VR-784	Not Detected
22	Human respiratory syncytial virus A	ATCC	VR-26	Not Detected
23	Human respiratory syncytial virus B	ATCC	VR-955	Not Detected
24	Parainfluenza 1 virus	ATCC	VR-1380	Not Detected
25	Human parainfluenza virus 2	ATCC	VR-92	Not Detected

NO.	Organism	Source	Isolate No.	Result†
26	Human parainfluenza virus 3	ATCC	VR-93	Not Detected
27	Human parainfluenza 4 virus 4a	ATCC	VR-1378	Not Detected
28	Human parainfluenza virus 4b	ATCC	VR-1377	Not Detected
29	Human Metapneumovirus (MPV)	KBPV	VR-87	Not Detected
30	Human adenovirus 1	ATCC	VR-1	Not Detected
31	Human adenovirus 11	KBPV	VR-63	Not Detected
32	Human adenovirus 18	ATCC	VR-1095	Not Detected
33	Human adenovirus 23	ATCC	VR-1101	Not Detected
34	Human adenovirus 3	ATCC	VR-3	Not Detected
35	Human adenovirus 4	ATCC	VR-1572	Not Detected
36	Human adenovirus 8	ATCC	VR-1368	Not Detected
37	Human adenovirus type 31	ATCC	VR-1109	Not Detected
38	Human adenovirus type 40	ATCC	VR-931	Not Detected
39	Human adenovirus type 5	KBPV	VR-61	Not Detected
40	Human adenovirus type 35	ATCC	VR-718	Not Detected
41	Human Bocavirus (HBoV)	Korean isolate		Not Detected
42	*Legionella pneumophila* Serotype 2	ATCC	33154	Not Detected
43	*Legionella pneumophila* subsp. fraseri Serotype 4	ATCC	33156	Not Detected
44	*Legionella pneumophila* Serotype 7	ATCC	33823	Not Detected
45	*Legionella pneumophila* Serotype 10	ATCC	43283	Not Detected
46	*Legionella pneumophila* Serotype 11	ATCC	43130	Not Detected
47	*Legionella pneumophila* Serotype 12	ATCC	43290	Not Detected
48	*Legionella pneumophila* Serotype 13	ATCC	43736	Not Detected
49	*Legionella pneumophila* Serotype 14	ATCC	43703	Not Detected
50	*Legionella pneumophila* subsp. fraseri Serotype 15	ATCC	35251	Not Detected
51	*Mycoplasma pneumoniae*	ATCC	15293	Not Detected
52	*Streptococcus salivarius*	KCTC	5512	Not Detected
53	*Staphlococcus epidermidis*	KCCM	40416	Not Detected
54	*Haemophilus influenzae*	ATCC	51907	Not Detected
55	*Mycobacterium tuberculosis*	ATCC	25177	Not Detected

NO.	Organism	Source	Isolate No.	Result†
56	*Alphacoronavirus 1 (feline infectious Peritonitis Virus), 79-1146*	BEI	NR-49097	Not Detected
57	*Porcine Respiratory Coronavirus, ISU-1*	BEI	NR-48572	Not Detected
58	*Chlamydia pneumoniae*	ATCC	53592	Not Detected
59	*Streptococcus pneumoniae*	KCCM	40410	Not Detected
60	*Streptococcus pyrogenes*	ATCC	19615	Not Detected
61	*Bordetella pertussis*	ATCC	BAA-589	Not Detected
62	*Pneumocystis jirovecii* (PJP)	Korean isolate		Not Detected
63	Pooled human nasal wash*	Korean isolate		Not Detected
64	*Candida albicans*	KCCM	11282	Not Detected
65	*Pseudomonas aeruginosa*	ZMC	801908	Not Detected

† Specificity tests were repeated 3 times.
* No.63 was used to evaluate the specificity of diverse microbial flora in the human respiratory tract.
※ ATCC: American Type Culture Collection,
BEI: BEI Resources
KBPV: Korea Bank for Pathogenic Viruses
ZMC: ZeptoMetrix Corporation
KCTC : Korean Collection for Type Cultures

2. Sensitivity

In order to determine the sensitivity of Allplex™ SARS-CoV-2 Assay, genomic RNA from SARS-CoV-2, obtained from TWIST BIOSCIENCE (Cat. No. 102024) and BEI Resources (Cat. No. NR-52286) was serially diluted into negative sample matrix. Nucleic acids were extracted from each dilution using Microlab NIMBUS IVD and analyzed with Allplex™ SARS-CoV-2 Assay. Detection limit of Allplex™ SARS-CoV-2 Assay verified using genomic RNA of TWIST BIOSICENCE is 5000 copies/mL (= 50 RNA copies/rxn). Detection limit of Allplex™ SARS-CoV-2 Assay verified using inactivated SARS-CoV-2 virus of BEI Resources is 1,000 viral genome equivalents/mL (= 1.0 GE/μL).
In order to determine the sensitivity of Allplex™ SARS-CoV-2 Assay for extraction-free method, inactivated SARS-CoV-2 virus of BEI Resources was roughly extracted with extraction-free method. Detection limit for extraction-free method is 10,000 viral genome equivalents/mL (= 10 GE/μL).

3. Reproducibility

The reproducibility test was prepared including High Negative (0.1 X LoD), Low positive (1XLoD) and Moderate positive (3XLoD) samples. At each testing site, the kit was tested for five days, two runs per day by two different experimenters and triplicate of each target. The positive rates were observed for each target for reproducibility study: 100.0% for Moderate positive samples, ≥95% for Low positive samples. The reproducibility of the Allplex™ SARS-CoV-2 Assay was evaluated between runs, sites and product lots. Positive rates for all concentrations and CV values met criteria of less than 10 (<10).
The results were satisfied with the Criteria set above, thus confirming the reproducible performances of Allplex™ SARS-CoV-2 Assay.

4. Interfering substances

There were no effects on the results by adding the substance: non-specific detections or inhibitions on target amplification. Based on the results, 7 interfering substances had no effect on Allplex™ SARS-CoV-2 Assay results.

No.	Interfering Substances	Source	Test Concentration
1	Mucin (bovine submaxillary gland, type I-S)	Sigma-Aldrich (Cat.No.M3895)	60 μg/ml
2	Mupirocin (Antibiotic, nasal ointment)	Sigma-Aldrich (Cat.No.1448901)	6.6 mg/ml
3	Oxymetazoline (Afrin Nasal Spray)	Sigma-Aldrich (Cat.No.O2378)	15% (v/v)
4	Blood	Human	2% (v/v)
5	Tobramycin (Antibacterial, systemic)	Sigma-Aldrich (Cat.No.T4014)	4.0 μg/mL
6	Zanamivir (Anti-viral drug-Relenza)	Sigma-Aldrich (Cat.No.SML0492)	3.3 mg/mL
7	Oseltamivir (Anti-viral drug-Tamiflu)	Sigma-Aldrich (Cat.No.1479304)	25 mg/mL

5. Clinical performance

5-1. Standard extraction

A total of 187 specimens were included in this clinical performance. The specimens consist of 5 nasopharyngeal aspirate samples, 61 nasopharyngeal swab samples, 5 bronchoalveolar lavage samples, 59 oropharyngeal (throat) swab samples, and 57 sputum samples. Clinical performance of the Allplex™ SARS-CoV-2 Assay was evaluated through the comparison with other SARS-CoV-2 Real-Time RT-PCR Diagnostic Panel which is CE-IVD approved before. This comparison test is shown more than 95% rate of agreement in clinical sample. Therefore, it is confirmed that the quality of Allplex™ SARS-CoV-2 Assay is valid. The performance is summarized in table.

		CE-IVD Approved Comparator Result		
		Positive	Negative	Total
Allplex™ SARS-CoV-2 Assay	Positive	96	3*	99
	Negative	0	88	88
	Total	96	91	187

- ORA(Overall rates of agreement): 98.40% (95% CI: 95.38% to 99.67%)
- PPA(Positive Percent Agreement): 100% (95% CI: 96.23% to 100.00%)
- NPA(Negative Percent Agreement): 96.70% (95% CI: 90.67% to 99.31%)

* Sequencing and confirms true positive.

5-2. Extraction-free method

A total of 111 swab specimens were included in this clinical performance of extraction-free method. Extraction-free method was compared with standard extraction to prove the clinical validity of extraction-free method for Allplex™ SARS-CoV-2 Assay. This comparison test is shown more than 95% rate of agreement in clinical sample. Therefore, it is confirmed that the quality of extraction-free method for Allplex™ SARS-CoV-2 Assay is valid. The performance is summarized in table.

Allplex™ SARS-CoV-2 Assay		Standard Extraction		
		Positive	Negative	Total
Extraction-free method	Positive	78	0	78
	Negative	1*	32	33
	Total	79	32	111

- ORA(Overall rates of agreement): 99.1% (95% CI: 95.07% to 99.8%)
- PPA(Positive Percent Agreement): 98.7% (95% CI: 93.17 % to 99.78%)
- NPA(Negative Percent Agreement): 100% (95% CI: 89.57% to 100%)

* Sequencing and confirms true positive.

REFERENCES

1. J. Y. Chun. [High Multiplex Molecular Diagnostics.] Seegene Bulletin. (2012) 1: 1-4
2. D. H. Lee. [TOCE: Innovative Technology for High Multiplex Real-time PCR.] Seegene Bulletin (2012) 1: 5-10
3. Y. J. Lee, *et al*. [Single-channel multiplexing without melting curve analysis in real-time PCR] Scientific Reports (2014) 4:7439
4. J. Y. Chun*, et al*. [Dual priming oligonucleotide system for the multiplex detection of respiratory viruses and SNP genotyping of CYP2C19 gene.] Nucleic Acids Research. (2007) 35(6): e40
5. Gobalenya AE, Baker SC, Baric RS, de Groot RJ, Drosten C, Gulyaeva AA, et al. (March 2020). "The species Severe acute respiratory syndrome-related coronavirus: classifying 2019-nCoV and naming it SARS-CoV-2". Nature Microbiology. 5 (4): 536–544. doi:10.1038/s41564-020-0695-z. PMID 32123347. Archived from the original on 5 March 2020. Retrieved 3 March 2020
6. "Coronavirus disease named Covid-19". BBC News Online. 11 February 2020. Archived from the original on 15 February 2020. Retrieved 15 February 2020
7. Surveillance case definitions for human infection with novel coronavirus (nCoV): interim guidance v1, January 2020 (Report). World Health Organization. January 2020. hdl:10665/330376. WHO/2019-nCoV/Surveillance/v2020.1
8. "Healthcare Professionals: Frequently Asked Questions and Answers". United States Centers for Disease Control and Prevention (CDC). 11 February 2020. Archived from the original on 14 February 2020. Retrieved 15 February 2020
9. "About Novel Coronavirus (2019-nCoV)". United States Centers for Disease Control and Prevention (CDC). 11 February 2020. Archived from the original on 11 February 2020. Retrieved 25 February 2020
10. "CoV2020". GISAID EpifluDB. Archived from the original on 12 January 2020. Retrieved 12 January 2020
11. "WHO Director-General's opening remarks at the media briefing on COVID-19 - 11 March 2020". World Health Organization (WHO) (Press release). 11 March 2020. Archived from the original on 11 March 2020. Retrieved 12 March 2020
12. Wee SL, McNeil Jr. DG, Hernández JC (30 January 2020). "W.H.O. Declares Global Emergency as Wuhan Coronavirus Spreads". The New York Times. Archived from the original on 30 January 2020. Retrieved 30 January 2020
13. Chan JF, Yuan S, Kok KH, To KK, Chu H, Yang J, et al. (February 2020). "A familial cluster of pneumonia associated with the 2019 novel coronavirus indicating person-to-person transmission: a study of a family cluster". The Lancet. 395 (10223): 514–523. doi:10.1016/S0140-6736(20)30154-9.

PMID 31986261

14. Zhou P, Yang XL, Wang XG, Hu B, Zhang L, Zhang W, et al. (February 2020). "A pneumonia outbreak associated with a new coronavirus of probable bat origin". Nature. 579 (7798): 270–273. doi:10.1038/s41586-020-2012-7. PMC 7095418. PMID 32015507
15. Perlman S (February 2020). "Another Decade, Another Coronavirus". The New England Journal of Medicine. 382 (8): 760–762. doi:10.1056/NEJMe2001126. PMID 31978944
16. Benvenuto D, Giovanetti M, Ciccozzi A, Spoto S, Angeletti S, Ciccozzi M (April 2020). "The 2019-new coronavirus epidemic: Evidence for virus evolution". Journal of Medical Virology. 92 (4): 455–459. doi:10.1002/jmv.25688. PMID 31994738
17. Novel Coronavirus (2019-nCoV): situation report, 22 (Report). World Health Organization. 11 February 2020. hdl:10665/330991
18. Shield C (7 February 2020). "Coronavirus: From bats to pangolins, how do viruses reach us?". Deutsche Welle. Retrieved 13 March 2020
19. Hui DS, I Azhar E, Madani TA, Ntoumi F, Kock R, Dar O, et al. (February 2020). "The continuing 2019-nCoV epidemic threat of novel coronaviruses to global health – The latest 2019 novel coronavirus outbreak in Wuhan, China". The International Journal of Infectious Diseases. 91: 264–266. doi:10.1016/j.ijid.2020.01.009. PMID 31953166. open access

SYMBOLS

Key to symbols used in the manual and labels.

Symbol	Explanation
IVD	In vitro diagnostic medical device
LOT	Batch code
REF	Catalogue number
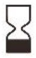	Use by date
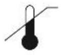	Upper limit of temperature
PRIMER	Oligonucleotide mix for amplification and detection
PREMIX	Enzyme mix
WATER	RNase-free Water
CONTROL +	Positive Control (PC)
CONTROL IC	Internal Control (IC)
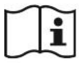	Consult instructions for use
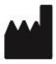	Manufacturer
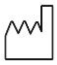	Date of manufacture
EC REP	Authorized representative in the European Community
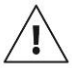	Caution
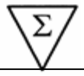	Contains sufficient for <n> tests

ORDERING INFORMATION

Cat. No.	Product	Size
**Allplex™ series**		
RV10247Y	Allplex™ SARS-CoV-2 Assay	50 rxns
**RV10248X**	**Allplex™ SARS-CoV-2 Assay**	**100 rxns***

* For use with the Microlab NIMBUS IVD, Microlab STARlet IVD, Seegene NIMBUS, and Seegene STARlet only

Cat. No.	Product	Size
**Accessory product**		
SG1701	Ribo_spin vRD (Viral RNA/DNA Extraction Kit)	50 preps
**Automated extraction systems**		
65415-02	Microlab NIMBUS IVD	EA
173000-075	Microlab STARlet IVD	EA
65415-03	Seegene NIMBUS	EA
67930-03	Seegene STARlet	EA
744300.4.UC384	STARMag 96 X 4 Universal Cartridge Kit	384 T / 1box
EX00013C	STARMag 96 X 4 Viral DNA/RNA 200 C Kit	384 T / 1box
SGprep32-180701	SGprep32	EA
EX00003P	STARMag 96 UniPlate	96 T / 1box
EX00004T	STARMag 96 UniTube	96 T / 1box
SG71100	SEEPREP32	EA
EX00009P	STARMag 96 ProPrep (Plate Type)	96 T / 1box
EX00009T	STARMag 96 ProPrep (Tube Type)	96 T / 1box
EX00017P	STARMag 96 ProPrep C (Plate Type)	96 T / 1 box
EX00017T	STARMag 96 ProPrep C (Tube Type)	96 T / 1 box
M9600	Maelstrom™ 9600	EA
W665S66	TANBead® Nucleic Acid Extraction Kit OptiPure Viral Auto Tube	72 T / 1box
W665A10	TANBead® Nucleic Acid Extraction Kit OptiPure Viral Bulk Plate	960 T / crt
